# α-Carbonic Anhydrase 1 Affects Photosynthetic CO_2_ Assimilation and Stomatal Conductance in *Arabidopsis thaliana* Plants

**DOI:** 10.3390/ijms27219575

**Published:** 2026-10-26

**Authors:** Natalia N. Rudenko, Maria Y. Ruppert, Gennady V. Mitenko, Elena M. Nadeeva, Marina A. Kozuleva, Lyudmila K. Ignatova, Boris N. Ivanov

**Affiliations:** 1 Institute of Basic Biological Problems, Federal Research Center, Pushchino Scientific Center for Biological Research of the Russian Academy of Sciences, Pushchino 142290, Russia; ms.maria1502@mail.ru (M.Y.R.); zhurikova-alena@yandex.ru (E.M.N.); kozuleva@gmail.com (M.A.K.); lkign@rambler.ru (L.K.I.); ivboni@rambler.ru (B.N.I.); 2 Institute of Physicochemical and Biological Problems in Soil Science, Federal Research Center, Pushchino Scientific Center for Biological Research of the Russian Academy of Sciences, Pushchino 142290, Russia; gen.mitenko@yandex.ru

**Keywords:** carbonic anhydrase, photosynthesis, *Arabidopsis thaliana*, carbon assimilation, chloroplast stroma, stomatal conductance

## Abstract

*Arabidopsis thaliana* plants of two mutant lines with a knockout of the *At3g52720* gene, encoding stromal α-carbonic anhydrase 1, αCA1 (αCA1-KO), and wild-type plants of the Columbia ecotype (Col), were grown under 450 ppm (NCO_2_) and 150 ppm (LCO_2_) CO_2_ in the air. The CO_2_ assimilation rate (ACO_2_) in plants grown under NCO_2_ was lower in the αCA1-KO plants than in Col plants at CO_2_ concentrations from 600 to 1200 ppm in the measurement chamber. In LCO_2_-grown plants, ACO_2_ values were lower than in NCO_2_-grown plants both in Col and mutants, with the same values in mutants and Col. Water use efficiency (WUE) in NCO_2_-grown mutant plants was lower than in Col at a CO_2_ concentration of 600 ppm and above, and in LCO_2_-grown mutants WUE became lower than in Col at 1200 ppm. The starch content was lower in leaves of mutants than in Col under both NCO_2_ and LCO_2_ conditions due to its reduced synthesis during the daytime photosynthesis. Under NCO_2_, the content of hydrogen peroxide was 55% higher in αCA1-KO plant leaves than in Col. Under LCO_2_, the content of H_2_O_2_ in Col increased to more than three times its level under NCO_2_, and became 2.5 times higher than in αCA1-KO, in which it decreased by 34% compared to that in NCO_2_ plants. Under NCO_2_ conditions, the contents of the stromal, cytosolic, and peroxisomal forms of ascorbate peroxidases (APXs) were about twice higher in αCA1-KO than in Col plants. Contrariwise, in LCO_2_-grown plants the contents of APXs were lower in mutants than in Col. The total protein amount, as well as the level of ribulose-1,5-bisphosphate carboxylase/oxygenase, were the same in Col and mutants under NCO_2_ conditions, but were 35% and 45–50%, respectively, lower in αCA1-KO than in Col under LCO_2_ conditions. In the leaves of NCO_2_-grown plants, the stomatal conductance was higher in αCA1-KO than in Col with slower stomatal opening and delayed closure in response to decreases and increases in CO_2_ level, respectively. At the same time, in the absence of αCA1, both the total number of stomata per unit leaf surface area, and the relative number of open stomata were higher than in Col leaves. In the protoplasts isolated from NCO_2_-grown αCA1-KO plants, the steady-state rates of oxygen evolution were 30% lower in mutants than in Col and were reached at a lower HCO_3_^−^ concentration (5 mM) than in protoplasts from Col (8 mM). The apparent half-saturating bicarbonate concentration was approximately 2 mM for αCA1-KO and 4 mM for Col. Our data demonstrate the involvement of the stromal chloroplast αCA1 in providing *A. thaliana* cells with CO_2_, and imply the significance of αCA1 in supplying inorganic carbon for starch and protein synthesis in plants. The increase in the total number of stomata per unit of leaf surface area and in the relative proportion of open stomata, as well as the reduced rate of stomatal responses in plants lacking αCA1, suggests the possible involvement of this enzyme in signaling processes regulating both stomatal movement and development.

## 1. Introduction

Growing food demand is putting significant pressure on agriculture as global crop production is predicted to double to meet the projected requirement driven by population growth, changes in dietary habits, and increased consumption of biofuels. The key factor determining the yield of agricultural crops is their capacity for photosynthesis. The latter is determined by the efficiency of CO_2_ conversion into biomass during its fixation in the Calvin–Benson–Bassham cycle (CBB cycle) in the carboxylation reaction of ribulose-1,5-bisphosphate (RuBP), which is the main process of carbon-assimilation reactions of photosynthesis, in which carbohydrate synthesis occurs. The uptake of inorganic carbon (Ci) from the air by higher terrestrial plants occurs via its diffusion into the leaves and, ultimately, into the chloroplasts; the limiting factor for carbon assimilation, especially under unfavorable conditions, is its insufficient supply to the carboxylation sites of RuBP in the chloroplast stroma [1,2,3].

Resistance to CO_2_ entry into the carboxylation sites arises from the stomatal pores, intercellular air spaces, the cell wall, the plasma membrane, the cytosol, the chloroplast envelope, and the chloroplast stroma. Along this pathway, repeated interconversions of Ci forms are required so that uncharged CO_2_ can cross hydrophobic barriers. Carbonic anhydrases (CAs) present in all living organisms are enzymes that catalyze the reversible hydration reaction of carbon dioxide with the formation of bicarbonate and a proton. In the genomes of higher plants of various species, there are up to several dozen, most often about 20, genes encoding CAs belonging to three families: α, β, and γ [4,5,6]. The first CA, located in the mesophyll cells of *Arabidopsis thaliana* leaves along the pathway of carbon dioxide supply to the carboxylation centers, is βCA4 (βCA4.1 isoform). Its presence was first demonstrated by the manifestation of its activity [7,8,9], and later it was identified by Fabre et al. [10] using the GFP-fusion method. This CA functions in a complex with the aquaporin PIP2;1 and promotes CO_2_ transport into cells [11]. Another isoform of this CA, βCA4.2, is located in the cytoplasm of photosynthetic cells, along with βCA2 [12], βCA3 [10], and the cytoplasmic form of βCA1 (βCA1.4), one of four isoforms of βCA1 originating from alternative splicing of the corresponding gene [13]. βCA2 and βCA4.2 are the most abundant cytoplasmic CAs in *A. thaliana*; their *ΔβCA4.2*/*βCA2* knockout led to significantly reduced growth under low CO_2_ conditions [12]. Ci is then transported into the chloroplast through the chloroplast envelope, where another isoform of βCA1 (βCA1.3) is located [13].

In the chloroplast stroma of various plants, the main forms of CAs are two isoforms of βCA1 (βCA1.1 and βCA1.2) [10]. The participation of βCA1 in the salicylic acid-induced stress signaling pathway was shown in a number of studies [14,15]. There is also evidence that βCA1 is able to play a role in the synthesis of ethylene, a phytohormone associated with plant growth inhibition [16]. The possible participation of βCA1 in enhancing Ci supply for photosynthesis, as well as its physiological role in general, has not yet been fully elucidated. The data demonstrating the necessity of this CA for photosynthesis were obtained after the knockout of βCA1 in *Oryza sativa* plants. Such mutants demonstrated decreased biomass and a lower net photosynthesis rate, despite the increased activity of ribulose-1,5-bisphosphate carboxylase/oxygenase enzyme (RuBisCO) [17]. Adult Arabidopsis plants with a knockout of the gene encoding βCA1 did not show reduced biomass or leaf size; however, our previous study showed that the CO_2_ assimilation rate in these plants under high light conditions was lower than in the wild-type (WT) [18], and the mutants also had lower CO_2_ content in chloroplasts, which led us to hypothesize that βCA1 accelerates the entry of Ci into chloroplasts. These mutants also demonstrated an increase in ΔpH on the thylakoid membrane under high illumination, apparently due to an increase in the pH of the chloroplast stroma [18]. In addition, a recent study has shown that βCA1 in *Solanum lycopersicum* plants functions as a buffer for light-induced changes in stromal pH, thereby optimizing the proton motive force and stabilizing photosystem II functioning [19].

The second stromal CA is βCA5 [10,20,21]. Its knockout in Arabidopsis has led to a significant suppression of growth and a decrease in plant fertility [15,21]. Tobacco Δ*βCA1*/*βCA5* mutants exhibited a reduced seed germination rate, decreased mass, and retarded growth compared to WT plants [20,22] due to a reduced rate of fatty acid synthesis, consistent with the role in fatty acid synthesis proposed for chloroplast CAs in 2002 [23].

αCA1 was discovered in the chloroplast stroma of *A. thaliana* using both the GFP-fusion method and microscopy with gold particles tagged with antibodies to αCA1 [24]. Later, its location in the chloroplasts was confirmed in a study of αCA1 (OsαCA1) from *O. sativa* [25]. Villarejo et al. [24] showed that this enzyme is transported into the stroma via a noncanonical pathway involving the Golgi apparatus. In this process, αCA1 undergoes post-translational modification by the addition of N-glycans. N-glycosylation was required for correct folding, trafficking and detectable enzymatic activity under the tested conditions [26]. Rice mutants with knocked out αCA1 (OsαCA1) demonstrated a significant reduction in the rate of photosynthesis, biomass accumulation, and grain yield compared to the WT, and had lower starch content in the leaves [25]. The absence of OsαCA1 in these plants led to a significant decrease in water use efficiency (WUE) compared to the WT. *A. thaliana* plants grown under high light also had lower starch content in the mutant leaves [18]. It is noteworthy that knocking out the *αCA1* gene in both rice and Arabidopsis led to an increase in stomatal conductance in these plants [18,25]. The *A. thaliana αCA1* knockout mutants exposed to increased illumination also had a reduced rate of assimilation, a slight decrease in the rate of electron transport and a decrease in ΔpH on the thylakoid membrane [18].

Besides the described function of CAs in mesophyll cells, an increasing amount of data has been accumulating regarding the role of CAs in supporting the development and functioning of stomata, whose guard-cell CAs are involved in controlling gas exchange between the atmosphere and leaves. First and foremost, this was demonstrated by the cooperative functioning of βCA4 and βCA1 [27,28,29]. Using GFP fusion, αCA7 was also demonstrated in the guard cells of *A. thaliana* stomata [30], where it plays the role of a sensor of the CO_2_ level in the air, participating in stomatal aperture size regulation and, consequently, in the regulation of transpiration. αCA7 was also shown to be involved in the formation of stomata in response to changes in the CO_2_ content of the air.

Despite the successes of recent years in identifying the locations and functions of CAs in higher plants in various metabolic pathways, their physiological role remains largely understudied. The main assumptions about the physiological role of the stromal CAs, which have been traditionally discussed in the literature, concerned their possible involvement in transporting Ci to the carboxylation sites, i.e., to RuBisCO, or in the conversion of bicarbonate, the predominant form of Ci in the alkaline stroma, into CO_2_, which is the substrate of the RuBisCO enzyme [31,32,33]. A three-dimensional reaction–diffusion model of photosynthesis in the mesophyll cell of C3 plants showed that, although the resistances of the cell wall and chloroplast envelope are the most significant limitations to photosynthesis, the level of CAs in the chloroplast stroma can also be a limiting factor for the rate of photosynthesis [34].

The aim of this work was to further investigate the possible participation of stromal αCA1 in Ci supply to carboxylation sites in higher plant chloroplasts and in stomatal conductance regulation. We compared plants grown at ambient and reduced levels of CO_2_, which is the main substrate of both photosynthesis and CA reaction.

## 2. Results

### 2.1. The Absence of αCA1 Affected Gas Exchange Parameters

Previously, we showed a significant increase in the CA activity of various cellular compartments, including the chloroplast stroma isolated from *A. thaliana* plants exposed to conditions of low CO_2_ level vs. normal and high CO_2_ conditions [35]. This indicates the importance of stromal CA activity in plant adaptation to low CO_2_ levels in the air. Therefore, αCA1 involvement in photosynthesis in *A. thaliana* plants was studied using αCA1-KO and Col plants adapted to a reduced CO_2_ content in the air (150 ppm, LCO_2_) for 14–16 days in comparison with plants grown at atmospheric CO_2_ (450 ppm, NCO_2_) (see Section 4.1).

**Figure 1:**
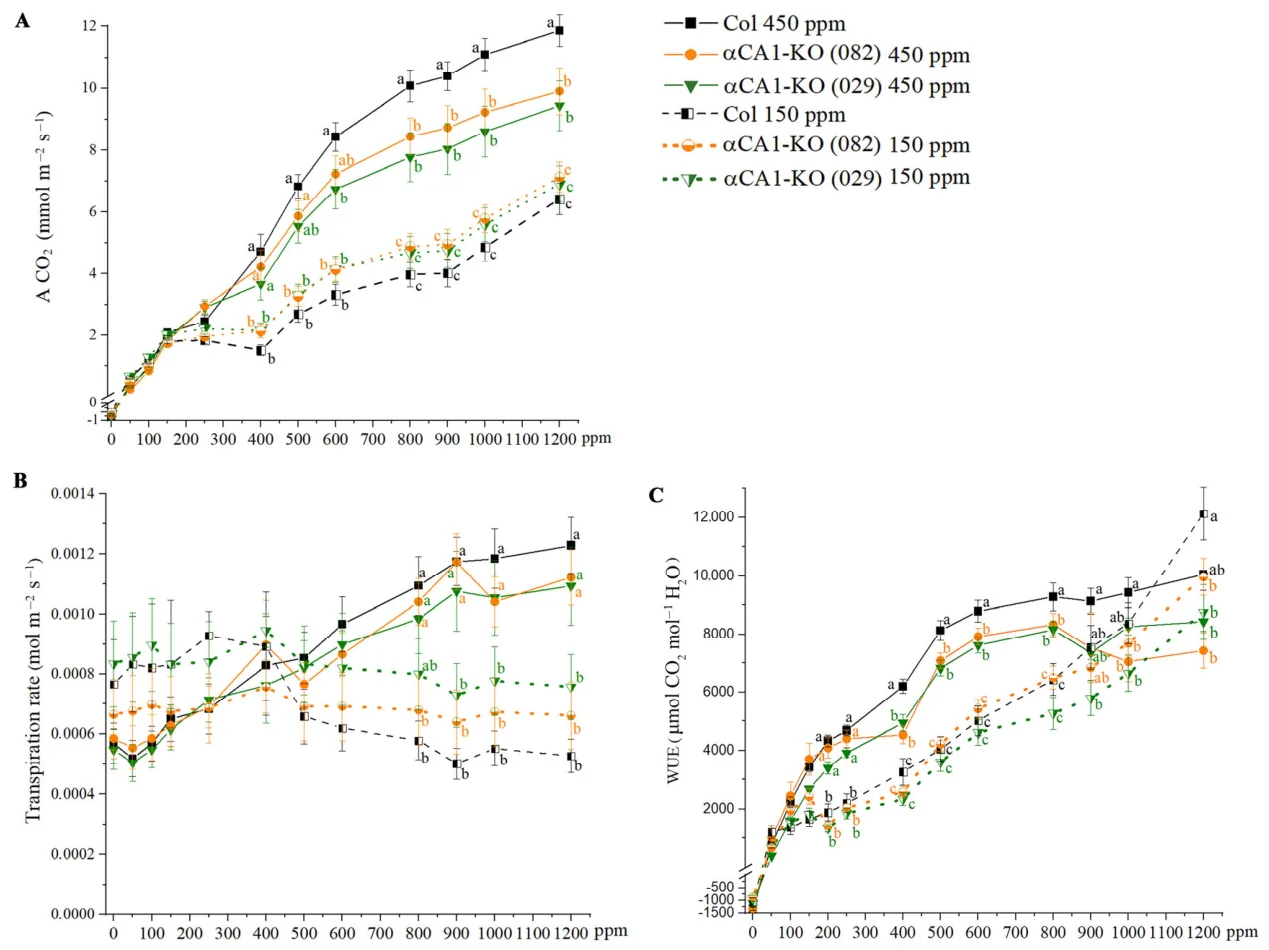
Dependence of CO_2_ assimilation rate (ACO_2_) (**A**), transpiration rate (**B**), and water use efficiency (WUE) (**C**) on the CO_2_ level in the measurement chamber in Col and αCA1-KO plants (082 and 029 lines) grown at 70–80 µmol quanta∙m^−2^∙s^−1^ under NCO_2_ (450 ppm CO_2_) and LCO_2_ (150 ppm CO_2_) conditions (see Section 4.1). The measurements were performed at 400 µmol quanta∙m^−2^∙s^−1^. More than 18 leaves from independent plants were analyzed for each genotype and CO_2_ growth conditions across four independent plant growth experiments (total *n* ≥ 18 of biological replicates across the four experiments). Measurements under different CO_2_ concentrations in the chamber represent repeated observations of the same biological replicate. Data are presented as means ± SD. Statistical significance of genotype and growth-CO_2_ effects was assessed by two-way ANOVA followed by the Holm–Bonferroni multiple-comparison test. Differences were considered statistically significant at *p* < 0.05. Letters near the symbols indicate statistically significant differences.

Gas exchange parameters in leaves were measured at carbon dioxide concentrations increased in steps from 0 to 1200 ppm. The CO_2_ assimilation rate (ACO_2_) in Col LCO_2_-grown plants became lower than in Col NCO_2_-grown plants at 400 ppm CO_2_ in the measurement chamber. These differences increased with increasing CO_2_ concentration, and ACO_2_ in LCO_2_ Col plants under 1200 ppm in the chamber became twice lower than in NCO_2_-grown Col plants (Figure 1A). ACO_2_ in plants grown under NCO_2_ was lower in the αCA1-KO plants than in Col plants at CO_2_ concentrations increased in the measurement chamber from 600 to 1200 ppm. ACO_2_ was lower in LCO_2_-grown plants of all three plant lines than in NCO_2_-grown plants with no statistically significant differences between LCO_2_-grown αCA1-KO and Col plants regardless of the concentration of carbon dioxide in the measurement chamber.

The transpiration rate (E) in all three plant lines grown at NCO_2_ increased as the CO_2_ concentration in the measurement chamber increased. In LCO_2_-grown Col plants, on the contrary, E decreased as CO_2_ increased in steps in the chamber (Figure 1B), but in LCO_2_-grown αCA1-KO this parameter was unchanged at any CO_2_ concentration in the measurement chamber. No significant differences in this parameter were observed between Col and mutant plants. Water use efficiency (WUE), which is the parameter reflecting the efficiency of water consumption in the process of carbon dioxide assimilation (ACO_2_/E), in NCO_2_-grown plants was lower in the mutants than in Col at 400 ppm CO_2_ and above. Although neither ACO_2_ nor E differed significantly between the mutants and Col in the LCO_2_-grown plants, WUE became significantly lower in αCA1-KO than in Col at 1200 ppm in the measurement chamber (Figure 1C).

### 2.2. Effects of αCA1 Gene Knockout on Arabidopsis Cell Redox Status

CO_2_ deficiency in plants results in elevated reactive oxygen species (ROS) formation [36,37] as a result of the Mehler reaction, i.e., O_2_ reduction by reduced cofactors of the photosynthetic electron transfer chain [38], and photorespiration, which is initiated when RuBisCO catalyzes the reaction of RuBP with O_2_ instead of CO_2_ [39,40].

We have previously demonstrated that under the control conditions of plant growth (under NCO_2_ and 70–80 µmol quanta∙m^−2^∙s^−1^) in αCA1-KO plants, the steady-state hydrogen peroxide level was higher than in Col plants [18]. In the present study, we have observed the same effect in NCO_2_-grown plants (Table 1). In αCA1-KO, 082 line, the H_2_O_2_ content was 55% higher than in Col. As expected, hydrogen peroxide levels increased in Col plants after adaptation to LCO_2_ conditions; however, the H_2_O_2_ level in αCA1-KO, 082 line, was 34% lower than in mutant plants under NCO_2_ conditions.

**Figure 2:**
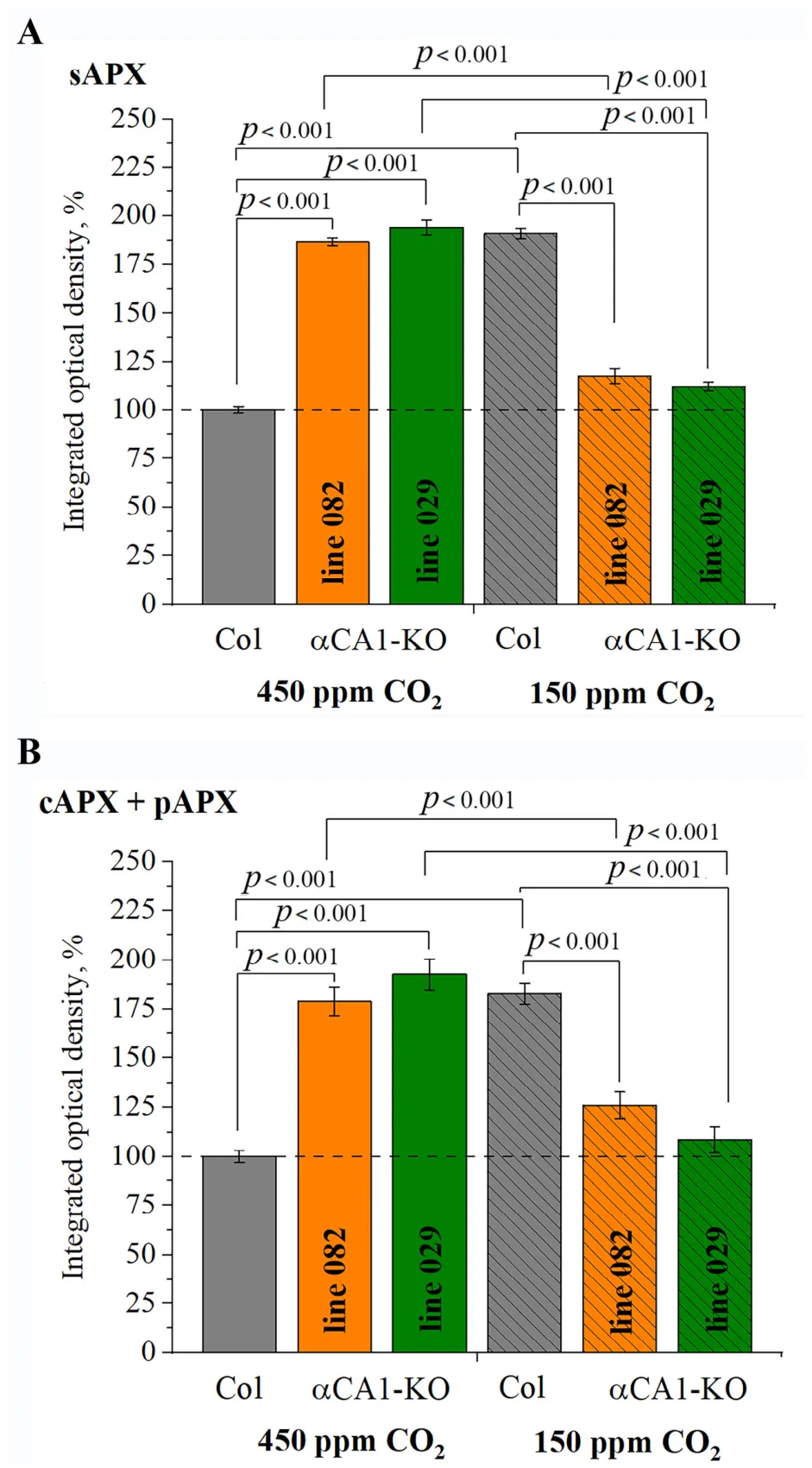
Abundance of ascorbate peroxidase (APX) isoforms in the leaves of WT *A. thaliana* (Col) and αCA1-KO plants (082 and 029 lines) grown under NCO_2_ (450 ppm CO_2_) and LCO_2_ (150 ppm CO_2_) conditions (see Section 4.1). Abundances were assessed as an integrated optical density (%) of immunoreactive bands corresponding to stromal APX (**A**) and peroxisomal APX with cytoplasmic APX (**B**). The optical densities of the bands with preparations from NCO_2_-grown Col with protein concentration of 15 μg were taken as 100%. Data are presented as means of normalized density values from three independent plant growth experiments ± SD. Statistical significance was determined by two-way ANOVA followed by the Holm–Bonferroni multiple-comparison test (*p* < 0.05). The figure presents the results from three independent experiments. The representative immunoblot images of a typical experiment are in Figure S1A.

The content of cytosolic, peroxisomal, and stromal isoforms of ascorbate peroxidase (APXs), which are important enzymes of the plant antioxidant system involved in H_2_O_2_ detoxification, was evaluated using immunoblotting of the total cell protein extract (Figure 2 and Figure S1). The bands of cytosolic (cAPX) and peroxisomal (pAPX) were quantified together as a combined signal. The content of thylakoid APX was not analyzed because the signal for this APX isoform was too weak (Figure S1). In NCO_2_-grown plants, the level of the cytoplasmic (cAPX), sAPX, and peroxisomal (pAPX) forms of APXs was about twice higher in αCA1-KO than in Col. Thus, changes in hydrogen peroxide content follow the same trend as changes in APX content in Col and mutant plants, depending on growth conditions. This was consistent with the content of APXs in Col plants under LCO_2_ conditions, which was also two-fold higher than in NCO_2_-grown Col plants. In LCO_2_-grown plants the contents of all forms of APXs were lower in mutants than in Col.

### 2.3. Starch Content and Transcript Abundance at the Beginning and End of the Light Period in αCA1-KO

**Figure 3:**
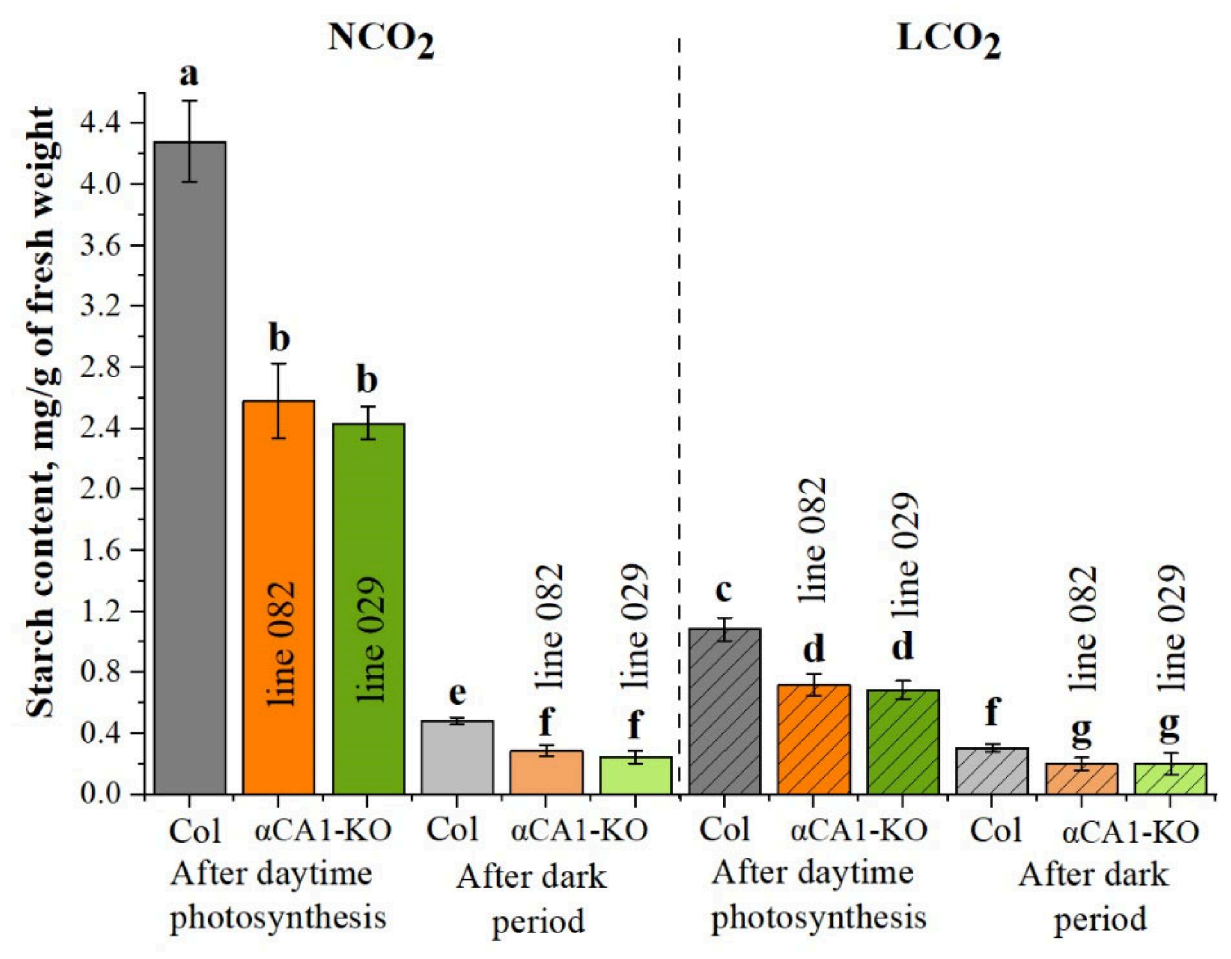
The starch content in leaves of *A. thaliana* Col and mutant plants with knocked out genes encoding αCA1 (lines 082 and 029) grown under NCO_2_ (450 ppm CO_2_) and LCO_2_ (150 ppm CO_2_) conditions. The starch content was measured at the beginning of the light period (after 16 h of darkness) and at the end of the light period (after 8 h of light). Data are presented as means of starch content in 6–7 plants ± SD. Statistical significance of genotype and growth-CO_2_ effects was assessed by three-way ANOVA followed by the Holm–Bonferroni multiple-comparison test. Differences were considered statistically significant at *p* < 0.05. Letters above the data indicate statistically significant differences between experimental variants. Data from a representative experiment are presented. Similar results were obtained in three independent plant growth cycles.

Starch is the main storage carbohydrate in plants, which accumulates in leaves during the photoperiod and is consumed during the night [41]. We have previously shown that starch content in the light in the leaves of plants grown under control conditions (under NCO_2_ and 70–80 µmol quanta∙m^−2^∙s^−1^) differs in the studied genotypes [18]. We further determined the starch content after 8 h of illumination, i.e., immediately before turning off the light and in the morning after a 16-h dark period, i.e., immediately after turning on the light (Figure 3).

After daytime photosynthesis, Col plants under LCO_2_ conditions accumulated 75% less starch than the same plants grown under NCO_2_ conditions (Figure 3). At the same time, the starch level was about 40% lower in αCA1-KO than in Col in both LCO_2_ and NCO_2_-grown plants.

Approximately 90% of starch in NCO_2_-grown Col and mutant plants was consumed overnight (Table 2). After adaptation to LCO_2_ conditions, Col plants consumed less starch (73%), while in αCA1-KO plants, starch consumption was the same as under NCO_2_. Consequently, after the dark period, the starch content became 40–50% lower in the mutants than in Col in both LCO_2_- and NCO_2_-grown plants (Figure 3). It can be concluded that the differences in starch content between αCA1-KO and Col grown under NCO_2_ conditions are primarily due to starch synthesis during daytime photosynthesis, whereas under LCO_2_ conditions, they are partly due to starch consumption during the dark period.

**Figure 4:**
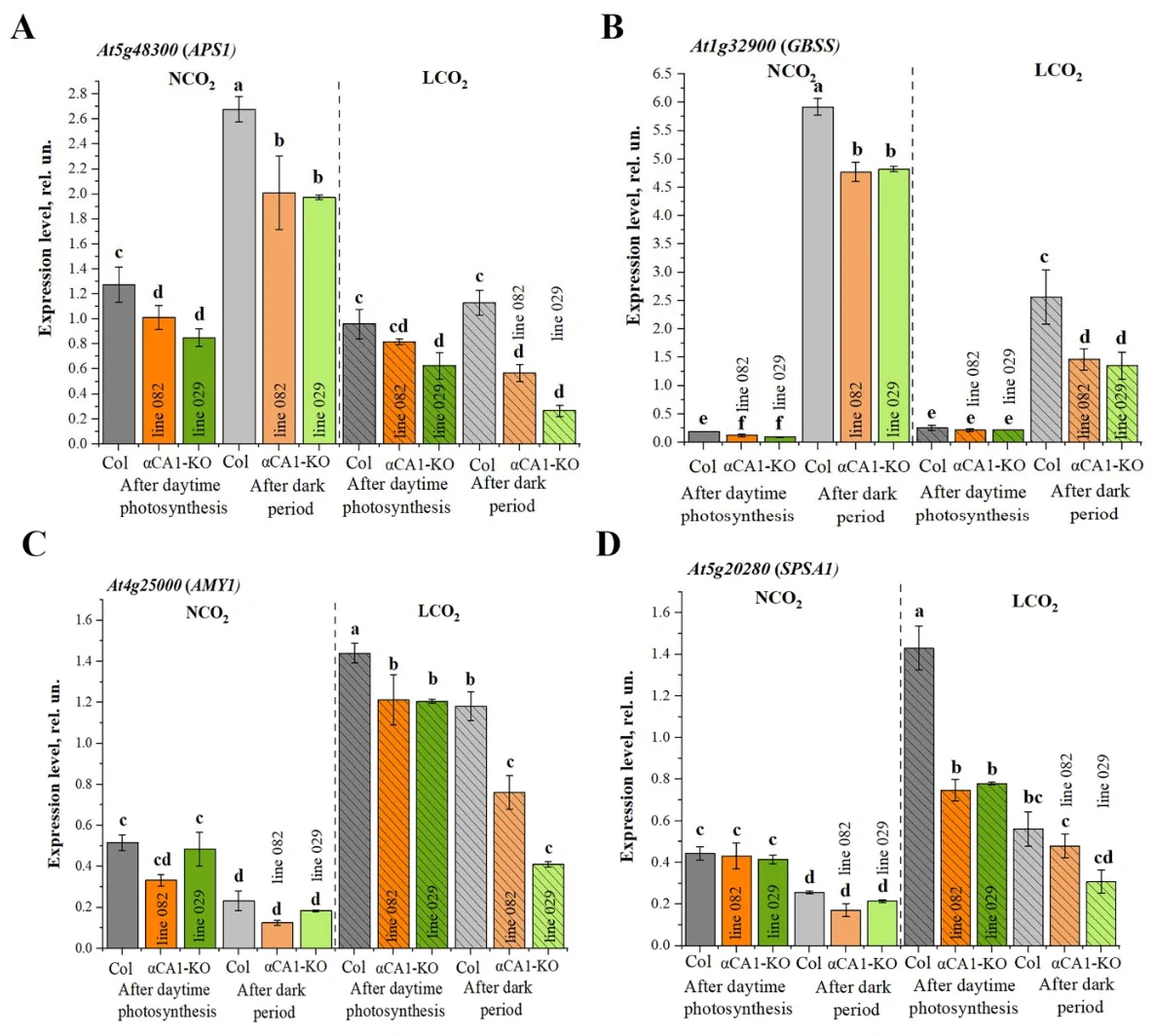
The expression level of the genes encoding the proteins involved in starch metabolism: ADP-glucose pyrophosphorylase (*APS1*) (**A**), granule-bound starch synthase 1 (*GBSS*) (**B**), endo-amylase α-amylase (*AMY1*) (**C**), and sucrose phosphate synthase 1 (*SPSA1*) (**D**) in the leaves of WT *A. thaliana* (Col) and αCA1-KO plants (082 and 029 lines) grown under NCO_2_ and LCO_2_ conditions. Leaf samples were collected after 16 h of darkness directly after turning on the light and after 8 h of light directly before turning off the light. Data were normalized for *Ubiquitin* gene expression. Values are means ± SD of three independent experiments with 3 plants sampled (total *n* = 9 across the three experiments). Statistical significance of genotype, growth-CO_2_ effects, and daytime was assessed by three-way ANOVA followed by the Holm–Bonferroni multiple-comparison test. Differences were considered statistically significant at *p* < 0.05. Letters above the data indicate statistically significant differences between experimental variants.

Further, we selectively analyzed the daily dynamics of the expression of several genes encoding the proteins involved in starch metabolism [41]. The leaves for RNA extraction were taken immediately after 8 h of illumination and immediately after turning on the light in the morning after a 16-h dark period. The expression of the genes encoding ADP-glucose pyrophosphorylase (APS1) and granule-bound starch synthase 1 (GBSS), enzymes involved in starch synthesis, showed a common trend in all three plant lines grown under NCO_2_- and LCO_2_ conditions: the expression level was the highest in the morning, namely at the beginning of the active photosynthetic period (Figure 4A,B), and decreased in the evening, at the end of plant photosynthetic activity. The expression levels of the analyzed genes, encoding enzymes involved in starch degradation metabolism—endo-amylase α-amylase (*AMY1*), sucrose phosphate synthase (*SPSA1*), phosphoglucan phosphatase starch excess 4 (*SEX4*), α-1,6-glucosidase starch debranching enzyme limit dextrinase (*LDA*), 4-α-glucanotransferase disproportionating enzyme (*DPE*), and sucrose phosphate synthase 4F (*SPS4F*)—were the highest in the evening, at the end of active photosynthesis, i.e., immediately before turning off the lights, and decreased in the morning, at the beginning of photosynthetic activity (Figure 4C,D and Figure S2).

Adaptation of plants to reduced CO_2_ led to an increase in the expression of *AMY1*, *SPSA1*, *PGM*, *LDA*, *DPE*, and *SPS4F* (Figure 4 and Figure S2). Such an increase was especially pronounced in the leaves at the end of daytime photosynthesis. In αCA1-KO, we observed a small but consistent decrease in the expression of four analyzed genes under NCO_2_ and LCO_2_ conditions, both after daytime photosynthesis and after the dark period: *APS1* (Figure 4A), *GBSS* (Figure 4B), *AMY1* (Figure 4C), *SPSA1* (Figure 4D). The expression levels of *PGI1*, *PGM*, *SEX4*, *LDA*, *DPE*, and *SPS4F* were similar in mutants and Col in both LCO_2_- and NCO_2_-grown plants (Figure S2).

### 2.4. Effects of αCA1 Gene Knockout on the Protein Content in A. thaliana Leaves

**Figure 5:**
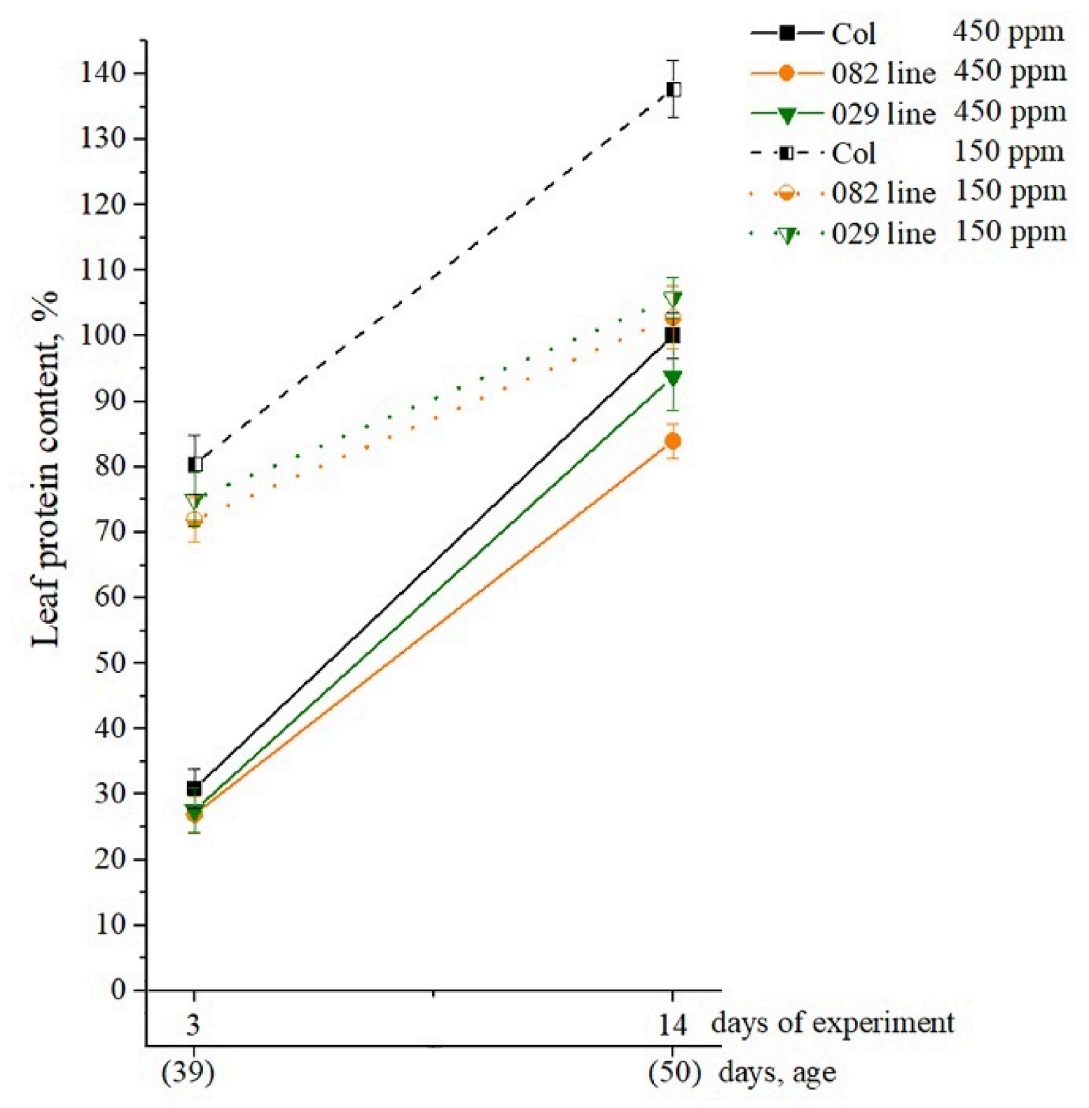
The total protein content in the leaves of the Col and αCA1-KO plants (082 and 029 lines). Leaf samples were collected from 39- and 50-day-old plants grown under conditions of NCO_2_ (450 ppm CO_2_) and from the plants of the same age exposed for 3 and 14 days to LCO_2_ (150 ppm CO_2_) conditions (see Section 4.1). The protein content (mg/g of fresh weight) in leaves of NCO_2_-grown Col plants at 50 days of age is taken as 100%. Averaged data from four experiments ± SD (%) are presented. Mutants were compared with Col under the same experimental conditions.

The total protein amount was measured in Col and mutant leaves at 39 and 50 days of age, in both NCO_2_- and LCO_2_-grown plants, which corresponded to the third and fourteenth days after exposure to LCO_2_ conditions (Figure 5). In NCO_2_-grown plants, protein content did not differ between Col and mutants and increased uniformly as plants grew from 39 to 50 days of age, corresponding to the vegetative phase of active plant growth. Just three days after exposure to LCO_2_ conditions, protein content increased significantly, by 50% in all three plant lines. By the 14th day of the experiment, the protein content continued to grow in approximately the same relative way in LCO_2_-grown Col plants, as well as in Col and αCA1-KO plants under NCO_2_ conditions. At the same time, mutants of both lines under LCO_2_ showed a significant decrease in the gain in protein content. Accordingly, by the 14th day under these conditions the protein content became approximately 35% lower than in LCO_2_-grown Col plants (Figure 5).

**Figure 6:**
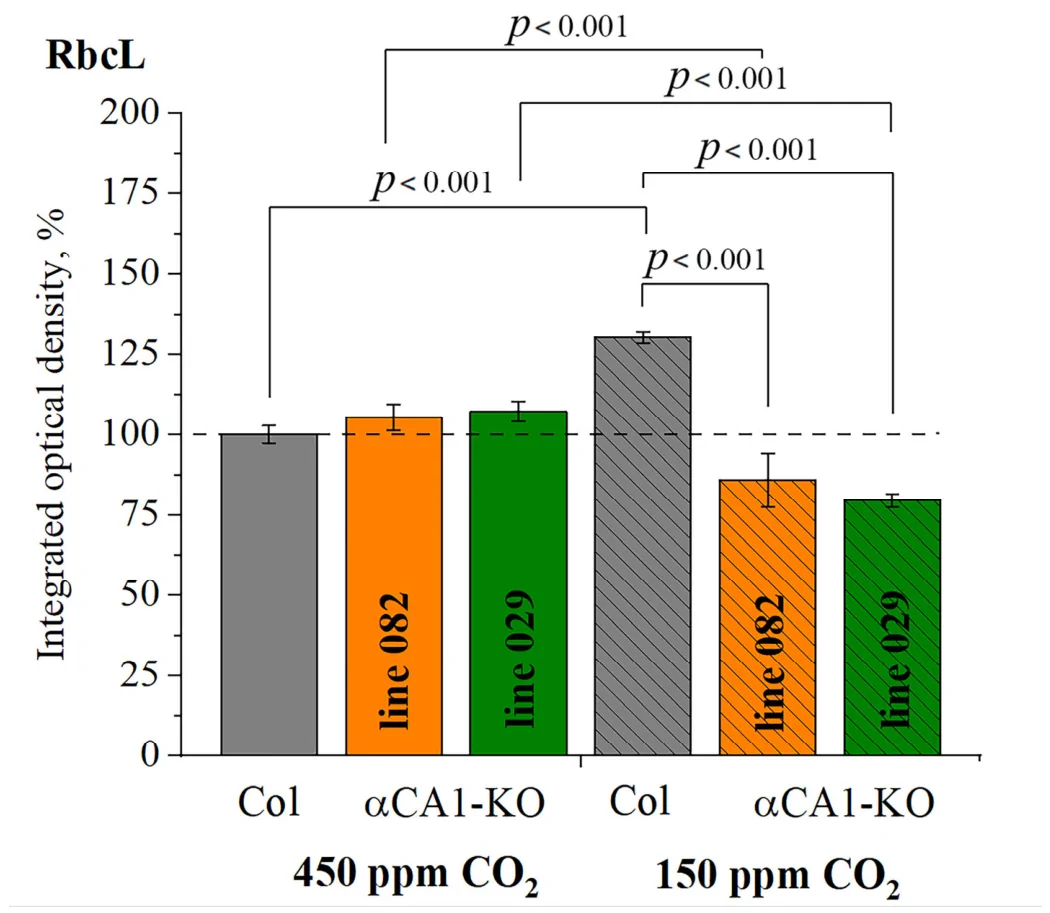
Abundance of the RuBisCO large subunit (RbcL) in the leaves of Col and αCA1-KO plants (082 and 029 lines) grown under NCO_2_ and LCO_2_ conditions. Abundances were assessed as an integrated optical density (%) of immunoreactive bands. The optical density of the band with preparations from NCO_2_-grown Col plants with protein concentration of 4 μg was taken as 100%. Data are presented as means of normalized density values from three independent plant growth experiments ± SD. Statistical significance was determined by two-way ANOVA followed by the Holm–Bonferroni multiple-comparison test (*p* < 0.05). The figure presents the results from three independent experiments. The representative immunoblot image of a typical experiment is in Figure S3.

In NCO_2_-grown plants, the level of the most abundant plant enzyme RuBisCO was the same in αCA1-KO and Col plants (Figure 6 and Figure S3). After two weeks under LCO_2_ conditions, the level of RuBisCO enzyme in Col plants increased 1.3-times compared with the same plants under NCO_2_ conditions. At the same time, in αCA1-KO plants, the RuBisCO level, in contrast, decreased by 20% in the 082 mutant line and 30% in the 029 line compared with the level of this enzyme in αCA1-KO plants in NCO_2_ conditions. Thus, the RuBisCO level became 45–50% lower in the mutants than in Col under LCO_2_ conditions. The decrease in RuBisCO level in LCO_2_-grown mutant plants vs. Col (Figure 6) was consistent with the decrease in total plant protein content in mutants compared to Col after 14 days of adaptation to LCO_2_ conditions (Figure 5).

**Figure 7:**
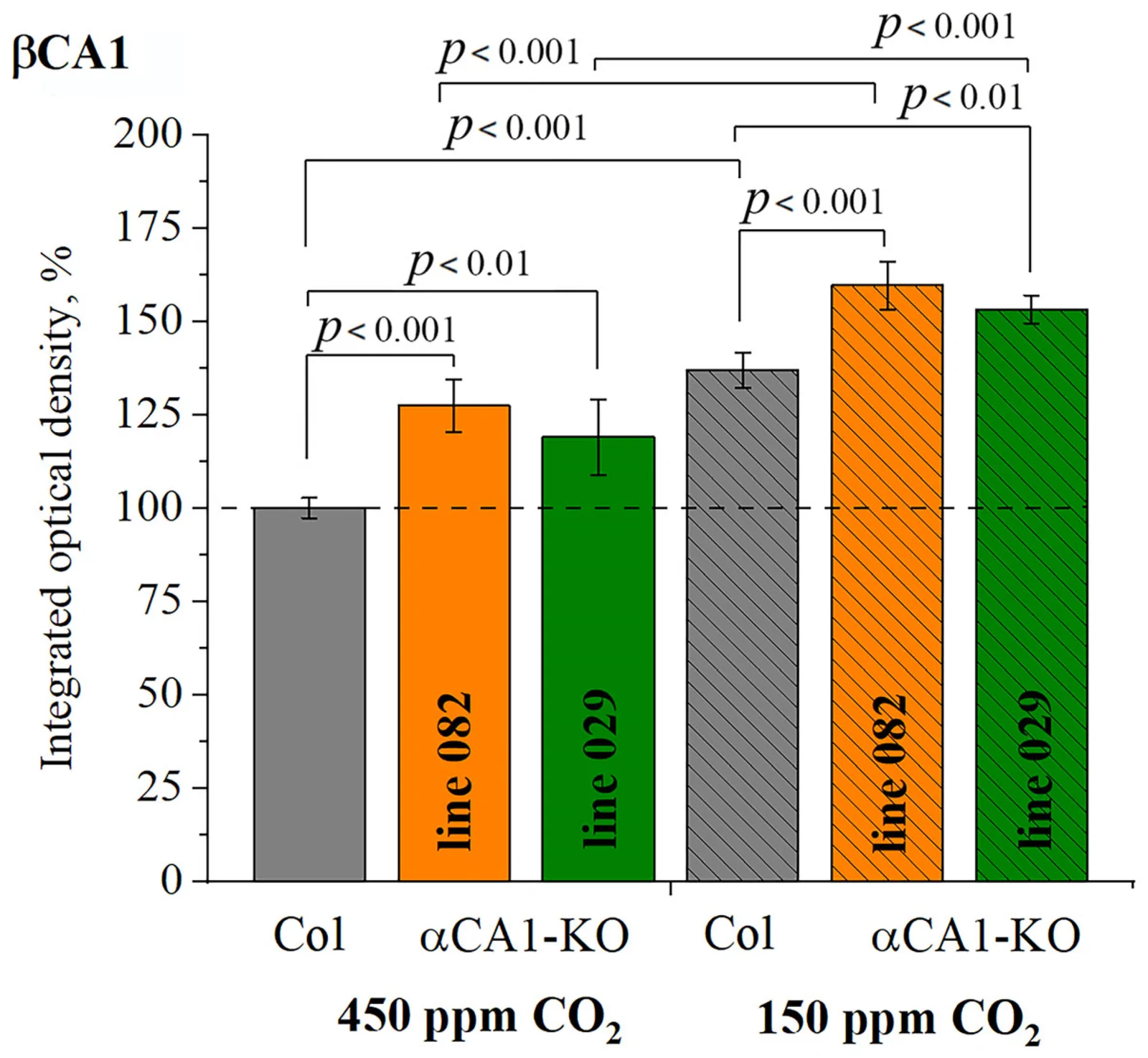
Abundance of βCA1 in the leaves of Col and αCA1-KO plants (082 and 029 lines) grown under NCO_2_ and LCO_2_ conditions (see Section 4.1). Abundances were assessed as an integrated optical density (%) of immunoreactive bands. The optical density of the band with preparations from NCO_2_-grown Col with protein concentration of 3 μg were taken as 100%. Data are presented as means of normalized density values from three independent plant growth experiments ± SD. Statistical significance was determined by two-way ANOVA followed by the Holm–Bonferroni multiple-comparison test (*p* < 0.05). The figure presents the results from three independent experiments. The representative immunoblot image of a typical experiment is in Figure S3.

Previously, we have shown that the *βCA1* gene expression level was increased in αCA1-KO vs. Col, which allowed us to assume that the mutant plants may partially compensate for the absence of αCA1 by increasing the intensity of βCA1 synthesis [18]. We examined the level of βCA1, which is also the second most abundant protein in plants after RuBisCO [42]. In NCO_2_-grown plants, the βCA1 level was about 20% higher in αCA1-KO than in Col plants (Figure 7 and Figure S3). After two weeks under LCO_2_ conditions, the βCA1 level in both Col and mutant plants increased by 30–40% compared with Col plants under NCO_2_ conditions and thus the content of this protein under LCO_2_ remained slightly higher, by 15–20% in mutants than in Col.

### 2.5. Stomatal Parameters in αCA1-KO and in Col Leaves

Data on differences between genotypes in the rate of CO_2_ assimilation by leaves and the intensity of starch formation suggest intracellular differences in the stages of Ci delivery to carboxylation centers in αCA1-deficient mutants. However, altered stomatal function may also contribute to the photosynthetic response to changes in CO_2_ concentration. We assessed the stomatal conductance, the number of stomata and the share of open stomata in Col and mutant plants.

**Figure 8:**
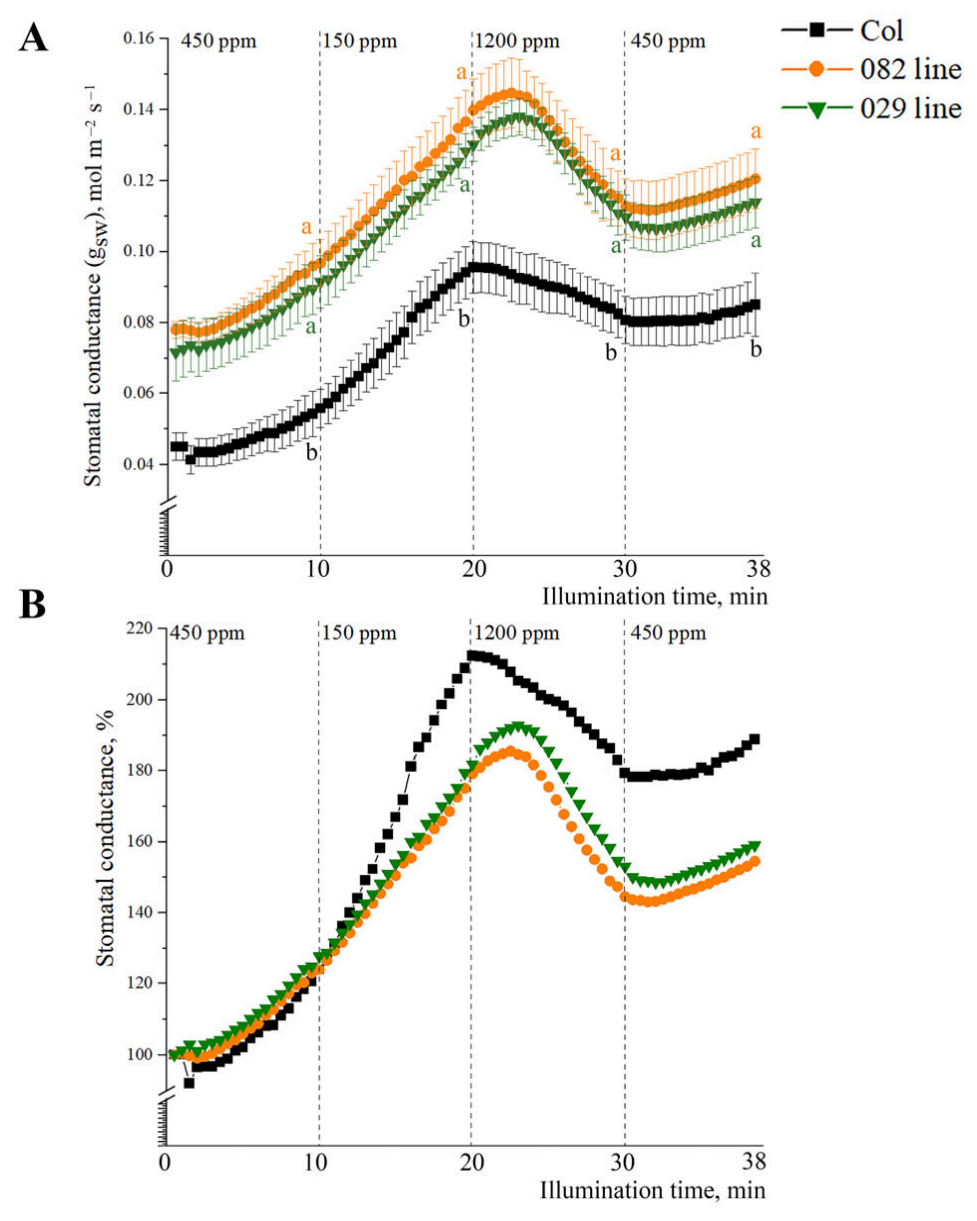
Stomatal conductance (g_sw_) dynamics in relation to CO_2_ concentration in the measurement chamber in Col and αCA1-KO plant leaves (lines 082 and 029) grown under NCO_2_ conditions, absolute values (**A**) and normalized to the initial value for each genotype (**B**). The differences were determined separately for each region of the curve corresponding to particular CO_2_ concentrations. The measurements were performed at 400 µmol quanta∙m^−2^∙s^−1^. Measurements at different chamber CO_2_ concentrations represent repeated observations of the same biological replicate. Data are presented as means of g_sw_ values at each time point of more than 18 leaves from independent plants from four independent plant growth experiments ± SD. Statistical significance of genotype and CO_2_ effects in the chamber was assessed by one-way ANOVA followed by the Holm–Bonferroni multiple-comparison test. The comparisons were performed separately at individual CO_2_ concentrations at equal time points from the moment of start of the measurements. Differences were considered statistically significant at *p* < 0.05. Letters near the symbols indicate statistically significant differences.

Stomatal conductance (g_sw_) was measured in dark-adapted Col and αCA1-KO plants (see Section 4.4) grown under control conditions (NCO_2_). In the leaves of all three plant lines, g_sw_ gradually increased after light onset—when measured at an ambient CO_2_ concentration of 450 ppm—and continued to rise after the CO_2_ concentration was lowered to 150 ppm (Figure 8A), reflecting an increase in stomatal aperture size [43,44]. Upon increasing the chamber CO_2_ concentration to 1200 ppm, g_sw_ declined in all three plant lines; however, in αCA1-KO leaves, unlike in Col, this decrease occurred with a slight time lag. Furthermore, the relative rate of stomatal opening in αCA1-KO plants increased slower than in Col lower after the CO_2_ concentration was lowered to 150 ppm (Figure 8B), and stomatal closure following the increase in chamber CO_2_ concentration to 1200 ppm began later than in Col. However, g_sw_ reduction for all three plant lines after 10 min at 1200 ppm CO_2_ reached 40% of the corresponding maximum values of this parameter. Thus, the knockout of αCA1 altered the kinetics of the stomatal response to changes in CO_2_ levels in the air.

**Figure 9:**
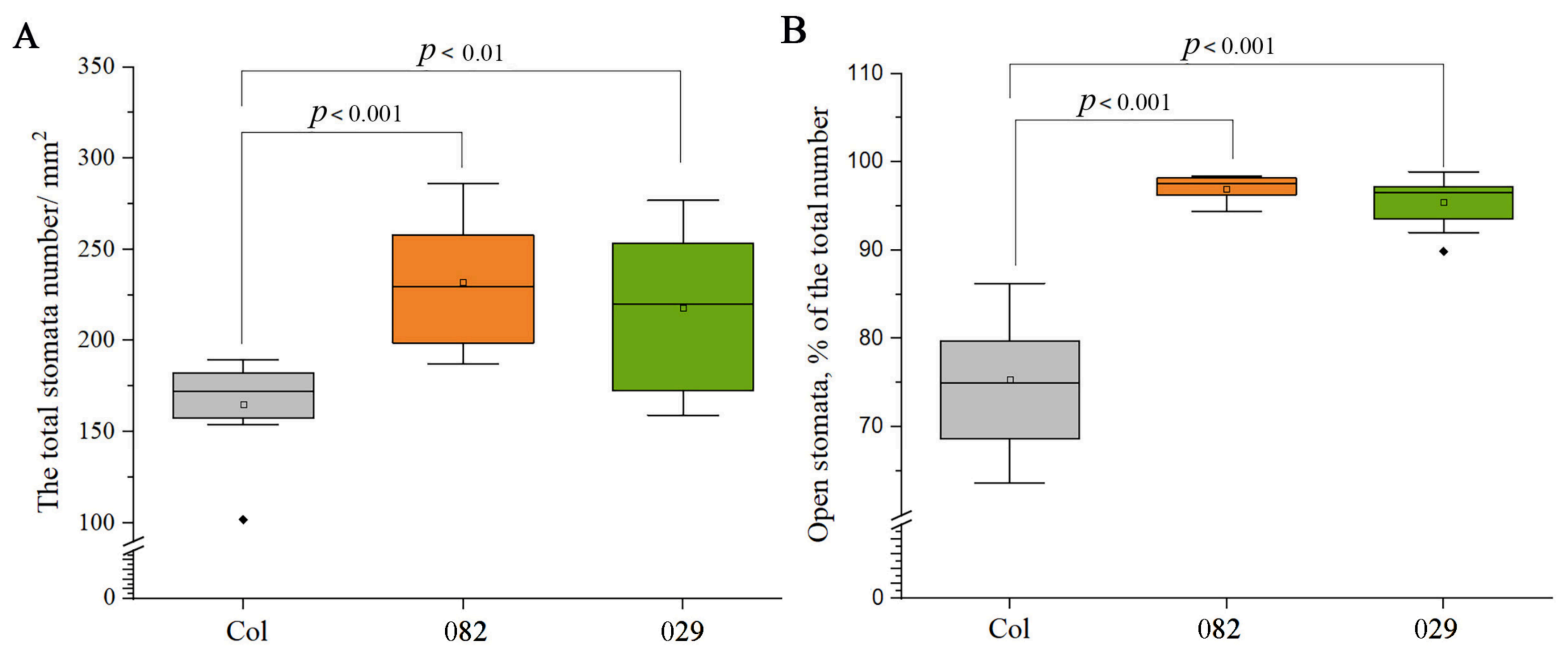
The total number of stomata calculated per 1 mm^2^ (**A**) and the number of open stomata of the total stomatal number, % (**B**) in Col and αCA1-KO (lines 082 and 029) plant leaves grown under NCO_2_ (see Figure S4). Data were obtained from four independent plant growth experiments. In total, at least 20 independent biological replicates were analyzed for each genotype across the four independent experiments. The middle part of the leaf of the middle layer, one leaf per plant were used for microscopy. Leaves were taken from 10 plants for each genotype, and two microscope fields per leaf were analyzed. Data are presented as means ± SD. Statistical significance among genotypes was assessed by one-way ANOVA followed by the Holm–Bonferroni multiple-comparison test. Differences were considered statistically significant at *p* < 0.05.

Microscopy of the leaf surfaces of NCO_2_-grown Col and mutant plants showed a 32% higher number of stomata per unit leaf area for both mutant lines than in Col (Figure 9A and Figure S4). At the same time, the share of open stomata was about 25% higher in αCA1-KO than in Col (Figure 9B and Figure S4). Thus, the higher stomatal conductance in αCA1-KO leaves (Figure 8A) is explained by both higher stomatal density and a greater proportion of open stomata on their leaf surface.

### 2.6. Photosynthetic Oxygen Evolution by Arabidopsis Mesophyll Protoplasts

**Figure 10:**
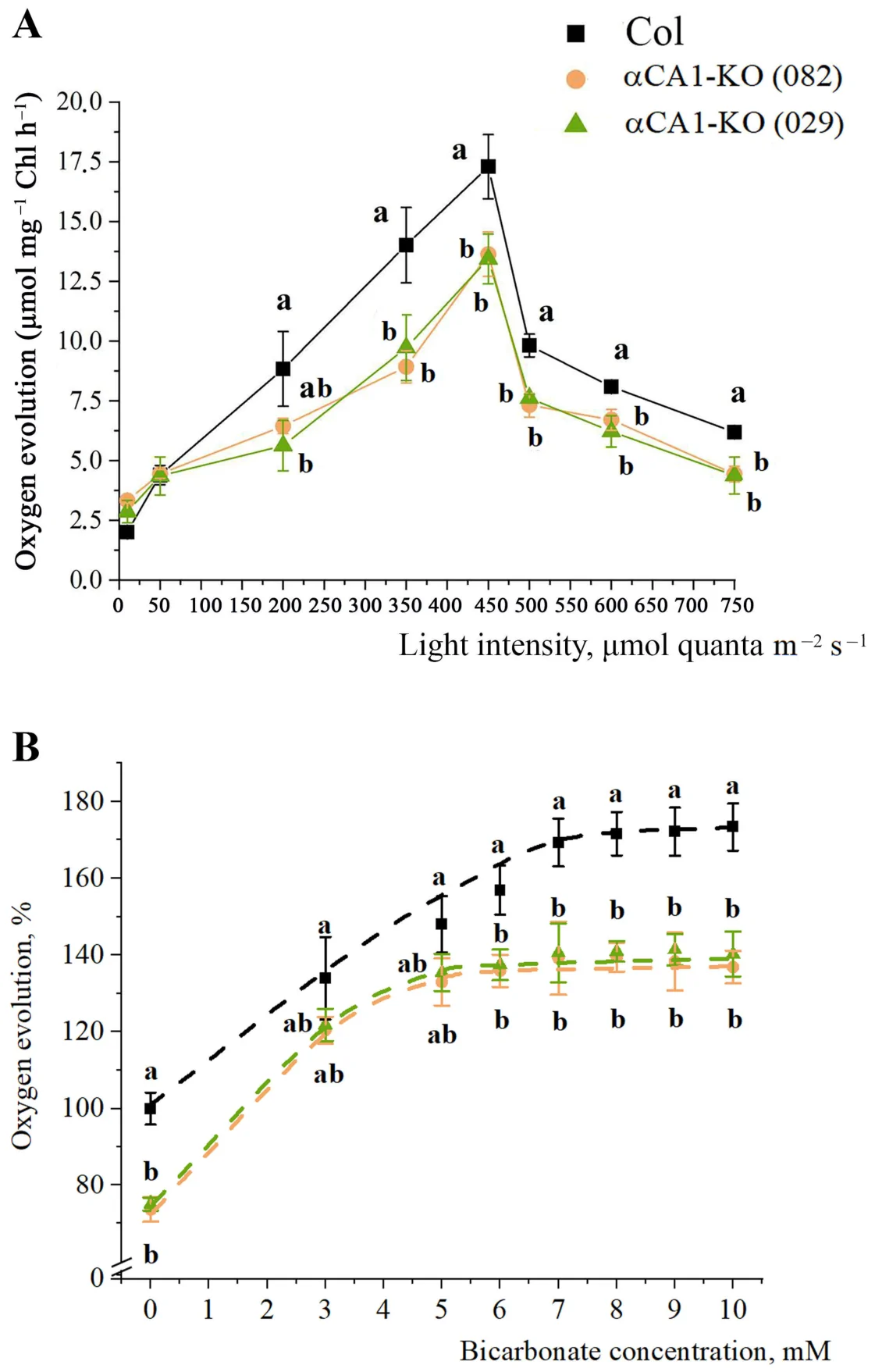
The dependence of oxygen evolution rate in protoplasts isolated from leaves of WT plants (Col) and αCA1-KO plants (082 and 029 lines) grown at 70–80 µmol quanta∙m^−2^∙s^−1^ under NCO_2_ conditions (450 ppm CO_2_) on light intensity (**A**) and bicarbonate concentration (**B**).

To study the dependence of photosynthetic efficiency on substrate concentration without the limitations for Ci transfer associated with stomatal functioning and transport across the cell wall, the photosynthesis rate as a function of Ci concentration was measured in protoplasts isolated from the leaves of Col plants and of the two αCA1-KO lines. The studies were carried out using protoplasts isolated from plants grown under control conditions (NCO_2_). In the first stage, the light intensity at which maximum protoplast activity was observed was determined. The dependence of the O_2_ evolution rate by protoplasts isolated from the leaves of Col and mutants on the light intensity was measured in a reaction medium purged with argon in the absence of HCO_3_^−^ and CO_2_ in the measurement cell. Already at a light intensity of 200 μmol quanta m^−2^ s^−1^, the O_2_ evolution rate by αCA1-KO protoplasts became approximately 30% lower than that by Col protoplasts (Figure 10A). These differences persisted until the maximum oxygen evolution rates at 450 μmol quanta m^−2^ s^−1^ were reached for the protoplasts of all three plant lines. With further increases in light intensity, the oxygen evolution rates decreased.

Further measurements of the O_2_ evolution rate in protoplasts, depending on bicarbonate concentration (added as a Ci source) in the medium, were carried out at a light intensity of 450 μmol quanta m^−2^ s^−1^, providing the maximum evolution rates. With increasing HCO_3_^−^ concentration, the rate of O_2_ evolution increased in protoplasts of all genotypes (Figure 10B). The steady-state rates of oxygen evolution were reached in the protoplasts from αCA1-KO at HCO_3_^−^ concentration of 5 mM and at 8 mM in protoplasts from Col with the apparent half-saturating bicarbonate concentration of 2.11 ± 0.26 mM for 082 line, 2.07 ± 0.21 for 029 line and of 3.74 ± 0.32 mM for Col (Figure 10B). At that, the rates of O_2_ evolution in Col protoplasts were 30% higher than in the mutant protoplasts at all HCO_3_^−^ concentrations studied. To quantify the bicarbonate dependence of O_2_ evolution, the resulting concentration–response curves were fitted with the Hill equation using fixed maximum and minimum O_2_ evolution rates for each genotype, allowing the apparent K_50_ values for bicarbonate to be determined.

(A) Measurements were performed without HCO_3_^−^ additions; 8 independent protoplast isolations performed from 15 to 20 leaves collected from three plants of each genotype per experiment. Oxygen evolution rate values from all experiments were used for statistical analysis (*n* ≥ 7).
(B) Oxygen evolution rate (%) by protoplasts as a function of bicarbonate concentration added to the measurement cell. The rate in Col protoplasts without HCO_3_^−^ addition was used as the 100% reference value. Measurements were performed at a light intensity of 450 µmol quanta∙m^−2^∙s^−1^; 12 independent protoplast isolations from 15 to 20 leaves collected from three plants of each genotype per experiment. Oxygen evolution rate values from all experiments were used for statistical analysis (*n* ≥ 7).

Statistical significance was assessed at *p* < 0.05 using one-way ANOVA followed by the Holm–Bonferroni multiple-comparison test. Data are presented as means ± SD. Letters near the symbols indicate statistically significant differences.

**Figure 11:**
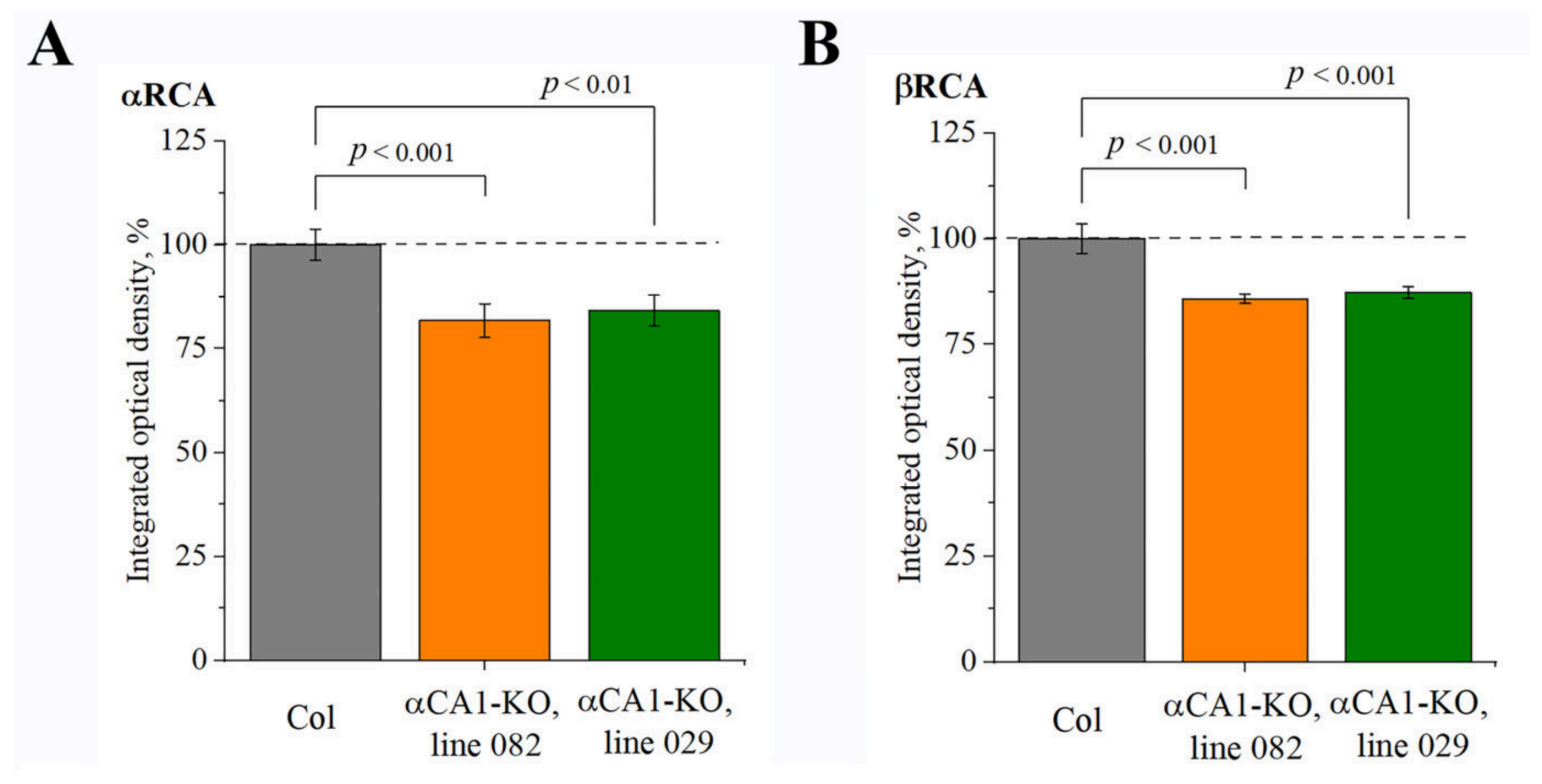
Abundance of RuBisCO activase (RCA), α (**A**) and β (**B**) isoforms, in the leaves of NCO_2_-grown Col and αCA1-KO plants (082 and 029 lines) assessed as an integrated optical density (%) of immunoreactive bands. The optical densities of the bands with preparations from Col with protein concentration of 4 μg were taken as 100%. Data are presented as means of normalized density values from three independent plant growth experiment ± SD. Statistical significance was determined by one-way ANOVA followed by the Holm–Bonferroni multiple-comparison test (*p* < 0.05). The figure presents the results from three independent experiments. The representative immunoblot image of a typical experiment is in Figure S5.

To investigate the reasons why αCA1-KO protoplasts required lower bicarbonate concentrations to achieve steady-state photosynthesis than Col protoplasts, we determined the levels of RuBisCO activase (RCA) in the corresponding plants. This protein is a central regulator of RuBisCO activity [45,46,47]. We found that in NCO_2_-grown αCA1-KO plants, the RCA content, of both α and β isoforms [48], was significantly lower, by 20%, than in Col (Figure 11).

## 3. Discussion

### 3.1. αCA1 Facilitates CO_2_ Supply and Photosynthetic Carbon Assimilation in Chloroplasts

CAs are widespread enzymes in living organisms, which have been “invented” at least eight times during evolution. These proteins perform a wide variety of functions in living organisms related to the supply or removal of Ci forms, pH regulation, and participation in stress signaling (see [12,49,50,51]). However, the physiological role of plant CAs, which has been actively studied for several decades, remains a largely unexplored issue. In the present study, we examined the possible participation of αCA1 in CO_2_ supply to carboxylation sites in Arabidopsis chloroplasts. Furthermore, our research was conducted using protoplasts from the same plants, which allowed us to investigate the dependence of photosynthetic efficiency without limitations imposed by carbon influx through stomata and transport through the cell wall. This approach also revealed a decrease in the efficiency of Ci assimilation in αCA1-KO vs. Col. The steady-state photosynthesis was achieved at lower HCO_3_^−^ concentrations in protoplasts isolated from αCA1-KO compared to protoplasts from Col, with the oxygen evolution rate in the mutants being 30% lower than in Col (Figure 10).

In NCO_2_-grown plants, the net photosynthesis rate, i.e., ACO_2_, in *A. thaliana* leaves increased with increasing CO_2_ concentration in the measurement chamber and the difference in this parameter between the mutants and Col plants increased with an increment in CO_2_ level (Figure 1A). These data are consistent with those of He et al. [25], who also showed a decrease in ACO_2_ in NCO_2_-grown rice plants with a knockout of the gene encoding OsαCA1, along with reduced WUE values in these plants. In our experiments, the transpiration rate did not differ significantly between αCA1-KO and Col (Figure 1B) with lower WUE values in αCA1-KO (Figure 1C).

An assumption of the reduced photosynthetic efficiency in plants with a lack of αCA1 due to the insufficient carbon dioxide supply to the carboxylation sites in chloroplasts is supported by the decreased starch content in αCA1-KO plants, both grown under NCO_2_ and under LCO_2_ (Figure 3). The decrease in starch content in mutant plants was accompanied by a slight decrease in the level of expression of two genes, *APS1* and *GBSS*, encoding enzymes involved in starch synthesis (Figure 4). APS1 is a component of the ADP-glucose pyrophosphorylase complex that catalyzes the formation of ADP-glucose, the activated glucosyl donor for starch biosynthesis [52,53], and GBSS is primarily responsible for amylose biosynthesis within the starch granule [41,54]. Although the reduction in starch content in αCA1-KO plants vs. Col (Figure 3) correlates with a decrease in the expression level of the specified genes in the mutants, such a slight reduction in the transcript level of only two genes is not the cause of this reduction.

Under NCO_2_ conditions, Col and both αCA1-KO lines consumed approximately 89–90% (Table 2) of their accumulated starch during the dark period; this indicates that the lower starch content observed in αCA1-KO plants at the start of the light period was largely due to the lower amount of starch accumulated during the preceding light period. Under LCO_2_, starch metabolism in the genotypes studied differed during both phases of the diurnal cycle. Although both αCA1-KO lines accumulated approximately 40% less starch during the light period (Figure 3) than Col, the proportion of starch they consumed at night was higher (84–85%, Table 2) than that of Col (73%). Such an increase in starch consumption during the night period in LCO_2_-grown mutant plants was accompanied by a slight but significant decrease in the expression level of two genes, *AMY1* and *SPSA1*, encoding enzymes involved in starch degradation in these plants, compared to Col (Figure 4). Thus, the changes observed in the mutants—in the rate of starch synthesis and also, though to a lesser extent, starch degradation—apparently reflect alterations occurring not at the transcriptional level but at the metabolic level; specifically, changes in enzyme activity, substrate availability, and the efficiency of utilizing carbon derived from starch. Several questions also remain regarding the precise contribution of αCA1 to CO_2_ supply within the chloroplast and its interaction with other CAs. Although our results support a role for αCA1 in facilitating the conversion of bicarbonate to CO_2_ and thereby supporting CO_2_ availability for RuBisCO, the extent to which this activity is functionally coordinated with other chloroplastic CAs and how it contributes to CO_2_ delivery under different inorganic carbon conditions remains to be established.

The participation of CAs in CO_2_ supply to the carboxylation sites in chloroplasts of higher plant cells may consist either in accelerating the interconversion of Ci forms during the transition between the aqueous and hydrophobic phases of organelles or in the conversion of HCO_3_^−^ into CO_2_, the direct substrate of RuBisCO in the chloroplast stroma. These assumptions were made several dozen years ago [31,32,33], but only recently these assumptions have begun to receive more substantial confirmations. A decrease in carbon assimilation rate in αCA1-deficient mutants has been shown [18,25] together with the data of the present study (Figure 1, Figure 2 and Figure 4). Moreover, the Cc value, which is a parameter reflecting the CO_2_ concentration in chloroplasts, was the same in αCA1-KO as in Col [18], indicating that the lower ACO_2_ values (Figure 1) in mutants were due to insufficient conversion of HCO_3_^−^ to CO_2_ in the stroma to ensure the CBB cycle, rather than a decrease in the rate of CO_2_ entry into chloroplasts. Furthermore, the lower ΔpH values observed in mutants compared to the WT in this study were presumably due to a shift in stromal pH toward more acidic values. This could have occurred due to a decrease in the rate of HCO_3_^−^ to CO_2_ conversion in the absence of αCA1, which, in turn, leads to a decrease in the rate of H^+^ consumption in the corresponding reaction [18]. The lower CO_2_ assimilation rates in intact αCA1-KO plants and lower oxygen-evolution rates in isolated protoplasts, together with reduced daytime starch accumulation and the results indicating an increase in photorespiration in mutants, imply that αCA1 is the CA that performs bicarbonate to CO_2_ conversion in the stroma for its use in the reaction catalyzed by RuBisCO.

### 3.2. Metabolic and Redox Consequences of αCA1 Deficiency

When CO_2_ supply in C3 plants is insufficient, for example, when stomata are closed, the CO_2_ assimilation rate is reduced [35], leading to increased ROS formation in various cellular compartments, primarily in chloroplasts, where the Mehler reaction takes place [55,56]. Furthermore, under conditions of limiting CO_2_ supply, RuBisCO oxygenation is activated [57], leading to increased photorespiration and the formation of ROS, specifically H_2_O_2_, primarily generated in the peroxisomes when the enzyme glycolate oxidase converts glycolate to glyoxylate [39,40]. In our experiments, hydrogen peroxide content was indeed increased in Col LCO_2_-grown plants vs. NCO_2_-grown ones (Table 1), as were the levels of three isoforms of APXs (Figure 2 and Figure S1). It is important that the levels of hydrogen peroxide and APXs were also elevated in αCA1-KO plants under NCO_2_, implying a higher rate of photorespiration and Mehler reaction in the mutant plants. These data also support the involvement of αCA1 in supporting CO_2_ fixation processes during its photosynthetic assimilation, which appears to be impaired in the absence of this enzyme, leading to increased photorespiration in these plants.

In plants grown under LCO_2_ conditions, the levels of hydrogen peroxide and APXs were lower in the mutants than in Col (Table 1, Figure 2 and Figure S1). A similar pattern of changes in hydrogen peroxide, as well as of the expression of the “stress marker” genes, after acclimation to another abiotic stress factor, i.e., to increased light intensity, has previously been observed in αCA1-KO leaves compared to Col [18]. We hypothesized that this effect can be explained by the greater activation of antioxidant systems in mutant plants under normal growth conditions and greater “readiness” of their antioxidant system for stress. This may explain a decrease in the level of hydrogen peroxide and such stress “markers” as antioxidant enzymes and transcripts of the stress-induced genes in response to such stress conditions as low CO_2_ level or increased illumination, instead of a further activation of the plant defense system.

Several questions remain regarding the mechanisms linking αCA1 deficiency to the observed changes in carbon metabolism, nitrogen balance, and redox homeostasis. In particular, it remains to be determined whether these metabolic and redox alterations arise primarily as a direct consequence of reduced chloroplastic CO_2_ availability or reflect secondary adjustments associated with changes in carbon assimilation and photorespiratory activity. Such studies may provide further insight into the role of αCA1 in coordinating photosynthetic carbon assimilation with cellular metabolic and redox homeostasis under changing CO_2_ conditions.

### 3.3. Protein Abundance and Possible Links to Carbon and Nitrogen Metabolism

CO_2_ from the air is not only a source of carbohydrates, but also one of the main Ci sources for protein biosynthesis. At that, protein content is also a measure of nitrogen storage. Our data demonstrate that under LCO_2_ conditions, carbon storage decreased. This was reflected in a reduction in starch in the mutants (Figure 3) with an increase in nitrogen storage, i.e., an increment of total protein amount (Figure 5). It is known that cellular carbon and nitrogen metabolism must be well coordinated to sustain optimal plant growth and development and that a significant part of the plant transcriptome is regulated by C/N balance (see [58,59]). The knockout of αCA1 under CO_2_-deficient conditions resulted in a smaller increase in overall protein level (Figure 5), as well as a reduction in the content of RuBisCO (Figure 6), which is the most abundant plant protein [60]. Furthermore, the absence of αCA1 altered C/N balance by the 14th day under LCO_2_. Thus, our data demonstrate the importance of αCA1 in providing *A. thaliana* cells with CO_2_, not only for carbohydrate synthesis in *A. thaliana* plants, but also for protein accumulation. There are also studies that showed that chloroplast CA activity was critical for supporting amino acid biosynthesis [6,22].

The level of the second most abundant plant protein, βCA1 [42], was slightly higher under both LCO_2_ and NCO_2_ in mutants than in Col (Figure 7), demonstrating that one form of CA compensates for the lack of another form of stromal protein, even under conditions that lead to the reduction in plant resources for protein synthesis. These data also confirm our findings on increased expression of the *βCA1* gene in αCA1-KO mutants vs. Col plants [18]. Besides, we have previously shown that the adaptation of Arabidopsis plants to LCO_2_ conditions led to a significant increase in the CA activity in preparations isolated from chloroplasts, with stromal CA activity increasing approximately threefold [35]. The increase in βCA1 abundance under LCO_2_ (Figure 7) is consistent with the evidence of the involvement of chloroplast CAs in acclimation to inorganic carbon limitation [35].

αCA1 knockout resulted in a decrease in the content of RCA (Figure 11). This protein is crucial for maintaining photosynthetic efficiency through CO_2_ assimilation under changing environmental conditions [61,62]. RCA acts as a catalytic chaperone, by releasing inhibitors such as ribulose-1,5-bisphosphate (RuBP) from the active site of RuBisCO. This allows a CO_2_ molecule to activate RuBisCO, enabling it to attach one more CO_2_ molecule to RuBP. Despite the lower RCA level in mutants compared to Col (Figure 11), the steady-state photosynthetic rates in mutants were achieved at lower Ci concentrations than in Col (Figure 10B).

### 3.4. αCA1 and Stomatal Regulation

Mutant plants partly compensated for a lack of αCA1 functioning by increasing the stomatal conductance (Figure 8A), likely due to a higher number of stomata per unit leaf area (Figure 9A) and a higher share of open stomata in αCA1-KO plants (Figure 9B), that was accompanied by an increase in g_sw_ in the leaves of mutant plants (Figure 8A). In recent years, more and more data on the participation of CAs in the regulation of stomatal conductance and in the transmission of external signals regulating stomatal aperture size have accumulated. It is important to note that some CA knockouts resulted in changed stomatal conductance vs. WT. This effect was determined by CA involvement in the signaling in the control of stomatal movement, as well as in a CA-mediated change in stomatal development. It is known that CA can take part in both the regulation of stomatal functioning and the regulation of their formation, acting as CO_2_ sensors in cells [27,63]. The indirect involvement of CA-mediated signaling in the control of stomatal movement, but not their development, was demonstrated in *Zea mays* plants with C4 photosynthesis [64]. Maize mutants with knockouts of the *ca1* and *ca2* genes demonstrated higher stomatal conductance than the WT and slower stomatal closure in response to increased CO_2_ partial pressure; however, these mutants did not show differences in stomatal density compared to the WT. The authors also noted changes in the rates of stomatal opening and closing in the mutants in response to changes in CO_2_ concentration during measurement. Similar results were obtained in our studies: plants lacking αCA1 demonstrated a slowed rate of stomatal response to changes in atmospheric CO_2_ concentration—specifically, a delayed closing response to a decrease in CO_2_ levels and a lag in the closing response to an increase in CO_2_ concentration (Figure 8). The participation of CAs in the regulation of gas exchange between the atmosphere and leaves has been studied most extensively for cooperatively functioning βCA4 and βCA1 [27,28,29]. The mechanism of their regulation of stomatal formation appears to be mediated by the extracellular propeptide Epidermal Patterning Factor 2 and the extracellular mediator of CO_2_-controlled stomatal development, the CO_2_ Response Secreted Protease [28]. The initial CA-catalyzed conversion of air CO_2_ to HCO_3_^−^ was shown to be critical, as *A. thaliana* ΔβCA1/βCA4 mutants were insensitive to elevated CO_2_ [27]. Stomatal responses to increased Ci operate through the intermediacy of abscisic acid [65,66,67]. Bicarbonate molecules, which are converted from CO_2_ by CAs in guard cells, activate anion channels. That process is mediated by kinases/phosphatases with participation of mitogen-activated protein kinases MPK4/MPK12, HT1 and CBC1 with the key kinase is considered to be OST1 [63,66,67]. The possibility of cooperative involvement of CAs in stomatal movement regulation was assumed for βCA1 together with βCA2, as well [68]. The ability to regulate transpiration by changing the size of stomatal aperture was also demonstrated for αCA7, which has recently been discovered in stomatal guard cells of *A. thaliana* [30]. This CA has been shown to participate in stomatal formation in response to changes in atmospheric CO_2_ content.

The data of the present study showed that αCA1 may also be involved in both the regulation of stomatal formation on the leaf surface of *A. thaliana* and the regulation of stomatal aperture size. Our findings are supported by the data of He et al. [25], who found higher stomatal conductance in rice mutants lacking OsαCA1 than in WT and suggested that the increased g_sw_ values were due to a feedback loop aimed at increasing CO_2_ uptake and compensating for the decreased CO_2_ availability in chloroplasts to minimize the reduction in carbon assimilation. Moreover, it is known that there is an inverse correlation between stomatal density and CO_2_ concentration in the air. The long-term cultivation of plants at elevated CO_2_ levels acts as a negative regulator of stomatal regulation in higher plants, leading to a decrease in the number of stomata on the leaf surface [69,70]. It can be assumed, therefore, that plants with *αCA1* knockout increase stomatal density (Figure 9A) in response to a CO_2_ deficit in supply for RuBisCO in the absence of this CA.

Several questions remain to be addressed regarding the mechanisms underlying the effects of αCA1 deficiency on stomatal development and stomatal aperture regulation. In particular, it remains to be determined whether the observed changes in stomatal characteristics and responses are mediated by direct CO_2_/Ci signaling or arise indirectly from altered chloroplastic CO_2_ availability and consequent changes in carbon metabolism.

## 4. Materials and Methods

### 4.1. Plants and Cultivation Conditions

The plants used in the study were *A. thaliana* WT plants, Columbia ecotype (Col) and plants with knockout of the *At3g52720* gene encoding αCA1 (αCA1-KO; homozygous lines 082 and 029) that were derived from the SALK_082033C and SALK_029393C lines, respectively. The plants were grown to the age of 36 days in a climate chamber at a light intensity of 70–80 µmol quanta·m^−2^·s^−1^ with an 8 h/16 h day/night photoperiod at the atmospheric CO_2_ concentration (450 ppm) at 19 °C. Next, some plants were left under the control conditions described above (NCO_2_), while the other plants were adapted to conditions of low CO_2_ level (150 ppm) in air (LCO_2_) for 14 days. The seeds of Col *A. thaliana* plants were kindly provided by Professor R. Scheibe from the Collection of the Plant Physiology Department at the University of Osnabrueck, Germany. The seeds of homozygous mutant plants were kindly provided by Professor J.V. Moroney (Louisiana State University, Baton Rouge, LA, USA). The location of the T-DNA insertion mutations in the *αCA1* gene is shown in Figure S6. The homozygosis status of the plants used in the work was confirmed using PCR (Figure S7). PCR with DNA from Col and with primers to *αCA1* gene showed a PCR product of the predicted size (lane 1 in Figure S7A). PCR with the same primers and DNA isolated from 029 and 082 mutant lines, lanes 6 and 7 in Figure S7A, respectively, showed the absence of a PCR product. PCR with cDNA synthesized from RNA isolated from Col and mutant plants and primers complementary to exon regions of *αCA1* gene showed the absence of transcripts of *αCA1* gene in 082 and 029 mutant plant lines (lanes 1 and 2 in Figure S7B, respectively) with the presence of transcripts of *αCA1* gene in preparations from Col (lane 3 in Figure S7B). The size of the PCR product in lane 3 corresponds to the predicted size.

### 4.2. Protoplast Isolation

The middle parts of 15–20 leaves from 6 to 7-week-old plants were excised and immediately cut into strips approximately 1 mm wide in extraction medium containing 5 mM MES–KOH (pH 5.5), 0.5 M sorbitol, 1 mM CaCl_2_, 0.2% bovine serum albumin (BSA), 5 mM benzamidine, 5 mM 6-aminocaproic acid, 5 mM Na_2_-EDTA, and 10 mM sodium ascorbate. Leaf strips were transferred to medium containing 5 mM MES–KOH (pH 5.5) with 0.5 M sorbitol, 1 mM CaCl_2_, 2% cellulase (Solarbio, Beijing, China), and 0.2% pectinase from *Aspergillus niger* (Honeywell Fluka, Seelze, Germany). Enzymatic digestion was carried out for 4 h at 28 °C under continuous illumination (80 µmol quanta·m^−2^·s^−1^) with gentle shaking on an orbital shaker at 80 rpm. Incubation was followed by the filtration of the obtained leaf suspension through an 80 μm nylon mesh. The filtrate was centrifuged at 30× *g* for 5 min, and the resulting pellet was resuspended in medium containing 5 mM MES–KOH (pH 6.2) with 0.5 M sorbitol and 1 mM CaCl_2_.

### 4.3. Measurement of Light-Induced Oxygen Evolution

The light-induced steady-state rate of photosynthetic O_2_ evolution was calculated from the rate of change in dissolved O_2_ concentration measured using a Clark-type oxygen electrode under continuous stirring at 21 °C. Measurements were performed in a reaction medium containing 20 mM HEPES–KOH (pH 7.6), 0.5 M sorbitol, and 1 mM CaCl_2_. Before measurements, the reaction medium was purged with argon for 30 min to remove dissolved CO_2_ and establish CO_2_-depleted conditions. The oxygen concentration was monitored continuously throughout each measurement. Following dark adaptation, the oxygen concentration was recorded in darkness until a stable linear trace was established. The slope of the oxygen concentration trace during this steady-state dark period was determined as the dark baseline rate. Samples were then illuminated with red light (660 nm), and illumination was maintained until the oxygen concentration trace reached a stationary phase. Upon illumination, a transient phase of net O_2_ uptake was initially observed, which is associated with the delayed activation of the CBB cycle and the resulting temporary imbalance between photosynthetic electron transport and downstream electron consumption. This initial transient phase was excluded from rate calculations. As the CBB cycle became activated and NADP^+^ regeneration increased, net O_2_ evolution progressively developed and reached a steady-state phase. The rate of light-driven photosynthetic O_2_ evolution was determined from the slope of the linear section of the oxygen concentration curve corresponding to the phase of O_2_ accumulation under illumination. The rate of light-induced photosynthetic O_2_ evolution was calculated from the slope of the linear portion of the oxygen concentration trace corresponding to this phase of O_2_ accumulation under illumination. The dark baseline rate was subtracted from the rate measured under illumination to obtain the net light-induced rate of photosynthetic O_2_ evolution. The oxygen concentration in the reaction chamber was determined from calibration data obtained using sodium dithionite as an oxygen scavenger. The calibrated oxygen content corresponded to the difference between electrode signal values recorded immediately before sodium dithionite addition and after complete oxygen depletion. Under the experimental conditions used (reaction medium containing 0.5 M sorbitol at 21 °C, 50% relative humidity, and atmospheric pressure of ~740 mmHg), the saturating oxygen concentration in the reaction medium was 0.250 μmol O_2_ mL^−1^. Light intensity was adjusted by varying the applied voltage and was measured as photon flux density (µmol quanta·m^−2^·s^−1^) using a LI-COR LI-250 quantum meter (LI-COR, Lincoln, NE, USA). Light-response curves were obtained at different light intensities (Figure 10A). The dependence of the O_2_ evolution rate on light intensity was determined in the absence of externally added HCO_3_^−^ or CO_2_. Light-response measurements were used to determine the light intensity corresponding to the maximum O_2_ evolution rate under CO_2_-depleted conditions (450 μmol quanta m^−2^ s^−1^). Subsequently, the dependence of the photosynthetic O_2_ evolution rate on HCO_3_^−^ concentration was determined at a light intensity of 450 μmol quanta m^−2^ s^−1^. HCO_3_^−^ was added to the reaction medium at the concentrations indicated in Figure 10B, using sodium bicarbonate (NaHCO_3_) as the source of bicarbonate ions.

### 4.4. Measurement of Gas Exchange in Plants

The CO_2_ assimilation rate (ACO_2_) and the transpiration rate (E) were measured in attached leaves using a portable photosynthetic apparatus (LI-6800, LI-COR, Lincoln, NE, USA) at a light intensity of 400 µmol quanta·m^−2^·s^−1^ (90% red light, 10% blue light) in a leaf chamber with CO_2_ changing in steps from 400 ppm to 0 ppm, then from 0 to 1200 ppm, and back to 400 ppm at 23 °C and 50% relative humidity. The equilibration time at each CO_2_ concentration was 300 s. The WUE was determined as the ACO_2_/E ratio.

Stomatal conductance (g_sw_) was measured in dark-adapted plants (1.5 h). After a saturating flash in the dark, the actinic light (400 μmol quanta∙m^−2^∙s^−1^) was turned on, followed by light flashes every 30 s. The measurements were made for 10 min at the CO_2_ concentration of 450 ppm in the chamber; then the concentration of CO_2_ was reduced to 150 ppm and the measurements were continued for another 10 min. Next, the CO_2_ concentration was increased to 1200 ppm for 10 min and then returned again to the initial value of 450 ppm for 8 min. The leaf area was measured with the Petiole mobile app version 26.09.04 (Petiole LTD, Peterborough, UK).

### 4.5. Starch and Protein Content Measurements

The content of starch was determined by measuring light absorption at 620 nm in the aqueous extracts of leaves after incubation with 0.12% KI [71]. The starch content was measured at the beginning of the light period and after 8 h of illumination. Crude protein extracts were prepared from *Arabidopsis thaliana* leaves according to the method described by Conlon and Salter (2007) [72], with modifications. Briefly, leaf tissue was extracted in E-buffer containing 125 mM Tris-HCl (pH 8.8), 1% (*w*/*v*) SDS, 60 mM Na_2_CO_3_, and 10% (*v*/*v*) glycerol, supplemented with a protease inhibitor cocktail (100×; Macklin, Shanghai, China). The extracts were incubated at 37 °C for 5 min and subsequently centrifuged at 12,000× *g* for 5 min using a MiniSpin centrifuge (Eppendorf, Hamburg, Germany). The resulting supernatants were used for protein quantification using the BCA Protein Assay Kit (Molecta, Moscow, Russia), based on the bicinchoninic acid method described by Smith et al. [73], according to the manufacturer’s instructions.

### 4.6. Hydrogen Peroxide Content

Hydrogen peroxide content was determined in leaves by the luminol-peroxidase reaction [74,75]. Leaves (50–100 mg) were taken from the growth chamber after 3 h of illumination, weighed and frozen in liquid nitrogen, transferred into 0.4 mL 2 M trichloroacetic acid, and homogenized. Hydrogen peroxide was extracted by adding 3 mL of 0.05 M K-phosphate buffer (pH 8.5). To remove chlorophyll and carotenoid molecules, the extract was incubated with 5% polyvinylpyrrolidone (PVP) and then centrifuged at 10,000× *g* for 10 min. The supernatant was collected and its pH was adjusted to 8.5 with 2 M KOH. Mixture of luminol (2.26 × 10^−4^ M) and peroxidase (1 × 10^−6^ M) (1 mL) was added to 50 µL of the extract directly in the measurement cuvette to determine the H_2_O_2_ level. Chemiluminescence was recorded with a Lum-100 luminometer (DISoft, Moscow, Russia) using solutions with known H_2_O_2_ concentration for calibration.

### 4.7. Reverse Transcription-Quantitative Polymerase Chain Reaction (RT-qPCR)

Total RNA was extracted from the frozen *Arabidopsis* leaves using the R-Plants kit (Biolabmix, Novosibirsk, Russia), and treated with DNase EM (Biolabmix, Novosibirsk, Russia) to eliminate any genomic DNA contamination. cDNA was synthesized using an RT-1 reverse transcription kit (Syntol, Moscow, Russia) using oligo(dT) as a primer in a LightCycler 96 Instrument (Roche Diagnostics GmbH, Rotkreuz, Switzerland). RT-qPCR was performed with a ready-to-use qPCRmix-HS SYBR reagent mixture (Evrogen, Moscow, Russia), using the primers for the genes encoding the proteins participating in starch synthesis and degradation (Table S1). Gene expression was calculated with the formula 2^−(*C*t sample–*C*t control)^, where *C*_t_ is the threshold number of PCR cycles. The resulting RT-qPCR data were normalized to the *Arabidopsis Ubiquitin* gene (*At5g25760*), which was the most stable according to BestKeeper version 1 [76], (SD 0.52, CV 2.16%).

### 4.8. Immunoblotting

Crude protein extracts from *A. thaliana* leaves were prepared using the modified extraction method described in Section 4.5, and protein concentration was determined according to the procedure described therein. Protein samples obtained were separated using denaturing electrophoresis by SDS-PAGE on 12% polyacrylamide gels following the protocol [77], in a Mini-PROTEAN Cell (Bio-Rad, Hercules, CA, USA). Protein extracts were diluted in a loading buffer containing 200 mM Tris (pH 6.8), 2% (*w*/*v*) SDS, 0.005% (*w*/*v*) bromophenol blue, and 5% (*w*/*v*) dithiothreitol. Samples were heated to 95 °C for 5 min and centrifuged at 12,000× *g* for 5 min in a MiniSpin centrifuge (Eppendorf, Germany). The supernatants were then loaded onto gels in amounts corresponding to 1–25 μg of total protein per lane. Protein Marker RAV-10 (6.5–270 kDa; Biolabmix, Novosibirsk, Russia) was used as a molecular mass marker.

After electrophoresis, proteins were transferred from the gels to PVDF membranes with a pore size of 0.22 µm (Bio-Rad, Hercules, CA, USA) using a Mini Trans-Blot Cell (Bio-Rad, Hercules, CA, USA). The membranes were then incubated in Tris-buffered saline (TBS) containing 20 mM Tris (pH 7.6) and 150 mM NaCl for 5 min, followed by blocking for 60 min in TBS containing 2% (*w*/*v*) nonfat dried milk. The membranes were then incubated with primary polyclonal rabbit antibodies against β carbonic anhydrase 1 (AS19 4321)*,* L-ascorbate peroxidases (AS08 368), the large RuBisCO subunit (AS15 2955), and RuBisCO activase (AS22 4868) (Agrisera Co., Vännäs, Sweden), washed in TBS, and incubated with secondary goat anti-rabbit IgG antibodies (Agrisera Co., Vännäs, Sweden). Protein bands were visualized using an alkaline phosphatase conjugate substrate kit (Bio-Rad, Hercules, CA, USA). Membranes were scanned using a transmission flatbed scanner (Epson V700, Suwa, Japan) in a high dynamic range mode with 16 bits per pixel.

Densitometric analysis of the Western blot results was performed to quantify the protein content in each lane by measuring the integrated optical density (IOD) of the protein bands using Fiji (a batteries-included distribution of ImageJ, version 1.54) and OriginPro 2021, version 9.8 software. Calibration curves were generated for each individual blot for each analyzed protein and its respective isoforms and are presented in the Supplementary Materials (Figures S1B,C, S3B,D and S5B,C).

### 4.9. Measurement of Stomatal Parameters

Fully expanded leaves from plants grown under NCO_2_ conditions were collected and cut into pieces of approximately 0.6 cm^2^. Each piece was mounted directly on the special flat sample holder (12 mm Pin-stub, TED PELLA Inc., Redding, CA, USA). The samples were fixed to a sample stub with carbon tape (TED PELLA Inc., Redding, CA, USA) providing a conductive path to ground according to the TESCAN VEGA3 Scanning Electron Microscope Operator’s Manual [78]. The samples were then slowly (24 h) dried at room temperature in a desiccator. After drying, the samples were sputtered with a gold layer using the combined Quorum Q150R ES Sputter Coater system (KU Nanofabrication Facility, Overland Park, KS, USA) to create an electrically conductive surface, following the procedure for preparing non-conductive samples [79]. Microscopy was performed using a Tescan VEGA™ Scanning Electron Microscope (SEM) (Tescan, Brno, Czech Republic). The samples were placed in the SEM chamber. After the SEM operating parameters had been adjusted, several sections of the leaf surface were photographed, maintaining the same settings for each sample. A scanning electron microscope (SEM) was used, operating parameters included an accelerating voltage of 20 kV, a working distance of 15 mm, a high vacuum level of approximately 6.5 × 10^−2^ Pa, a magnification ratio of 467×, and a scale bar of 200 μm is shown in each image for verification of 200 μm. The stomatal density (SD) was calculated per 1 mm^2^ and the ratio of completely closed stomata to the total number of stomata in the visible field of the microscope was calculated.

### 4.10. Statistical Analysis

Statistical analysis was conducted using OriginPro software. Depending on the experimental design, statistical significance was assessed using one-way, two-way, or three-way analysis of variance (ANOVA), followed by the Holm–Bonferroni method for multiple comparisons, where applicable. Differences were considered statistically significant at *p* < 0.05.

## 5. Conclusions

This study provides evidence that stromal α-carbonic anhydrase 1 (αCA1) contributes to inorganic carbon supply to the sites of carboxylation in the chloroplast stroma of *Arabidopsis thaliana*. Our results suggest that αCA1 may contribute to photosynthetic carbon assimilation through two complementary processes: regulation of stomatal function and maintenance of CO_2_ availability for carbon fixation within the leaf cells, as supported by experiments with isolated protoplasts. However, the underlying mechanisms remain to be elucidated. These findings support the hypothesis that multiple carbonic anhydrases in C3 plants, which lack dedicated CO_2_ concentrating mechanisms, contribute to maintaining inorganic carbon supply to RuBisCO. Furthermore, αCA1 represents a potential target for genetic engineering aimed at improving photosynthetic carbon assimilation in C3 crops.

## Figures and Tables

**Table 1:** Hydrogen peroxide content in the leaves of the WT *A. thaliana* plants (Col) and αCA1-KO mutants (line 082) grown under NCO_2_ and LCO_2_ conditions. Leaves were sampled after 3 h of exposure to a growth light. For each genotype and CO_2_ level, three samples with 3–4 leaves were collected. Three analytical replicates were performed for each biological sample (*n* ≥ 9 per experiment). Data are presented as means ± SD. Statistical significance of genotype and growth-CO_2_ effects was assessed by two-way ANOVA followed by the Holm–Bonferroni multiple-comparison test. Differences were considered statistically significant at *p* < 0.05. Letters above the data indicate statistically significant differences between experimental variants. Four independent plant cultivation experiments were performed and showed the similar result.

CO_2_ Level, ppm	Plants	H_2_O_2_ Content, μmol H_2_O_2_/g of Fresh Weight
450 ppm (NCO_2_)	Col	0.74 ± 0.08 ^c^
	αCA1-KO (082 line)	1.35 ± 0.35 ^a^
150 ppm (LCO_2_)	Col	2.40 ± 0.70 ^a^
	αCA1-KO (082 line)	0.89 ± 0.19 ^b^

**Table 2:** The consumption of starch in the leaves of the WT *A. thaliana* plants (Col) and αCA1-KO mutants (lines 082 and 029) grown under NCO_2_ and under LCO_2_ (see Section 4.1). Data are presented as the means of starch content in 6–7 plants ± SD. Statistical significance of genotype and growth-CO_2_ effects was assessed by two-way ANOVA followed by the Holm–Bonferroni multiple-comparison test. Differences were considered statistically significant at *p* < 0.05. Letters above the data indicate statistically significant differences between experimental variants. Data from a representative experiment are presented. Similar results were obtained in three independent plant growth cycles.

CO_2_ Level, ppm	Plants	Starch Consumption Overnight, %
450 ppm (NCO_2_)	Col	88.8 ± 0.5 ^a^
	αCA1-KO (082 line)	88.8 ± 1.4 ^a^
	αCA1-KO (029 line)	89.9 ± 1.7 ^a^
150 ppm (LCO_2_)	Col	73.4 ± 2.1 ^b^
	αCA1-KO (082 line)	85.3 ± 4.3 ^a^
	αCA1-KO (029 line)	84.1 ± 3.9 ^a^

## Data Availability

The original contributions presented in this study are included in the article/Supplementary Material. Further inquiries can be directed to the corresponding author.

## Supplementary Materials

The following supporting information can be downloaded at: https://www.mdpi.com/article/10.3390/ijms27219575/s1.
