# Supplementary material for: α-Carbonic Anhydrase 1 Affects Photosynthetic CO2 Assimilation and Stomatal Conductance in Arabidopsis thaliana Plants
Source: Int J Mol Sci. 2026 Oct 26;27(21):9575. doi: 10.3390/ijms27219575 (PMC13762840; doi:10.3390/ijms27219575)
Supplement: Supplementary file 1 [file ijms-27-09575-s001.zip › ijms-4630828-supplementary.pdf]

Supplementary material

α Carbonic anhydrase 1 affects photosynthetic CO_2 assimilation and stomatal conductance in *Arabidopsis thaliana* plants

Natalia N. Rudenko[1]*, Maria Yu. Ruppert[1], Gennady V. Mitenko[2], Elena M. Nadeeva[1], Marina A. Kozuleva[1], Lyudmila K. Ignatova[1], and Boris N. Ivanov[1]

[1]Institute of Basic Biological Problems, Federal Research Center «Pushchino Scientific Center for Biological Research of the Russian Academy of Sciences» Pushchino, Moscow Region, Russia 142290

[2]Institute of Physicochemical and Biological Problems in Soil Science, «Pushchino Scientific Center for Biological Research of the Russian Academy of Sciences» Pushchino, Moscow Region, Russia 142290

*Correspondence: nataliacherry413@gmail.com

A

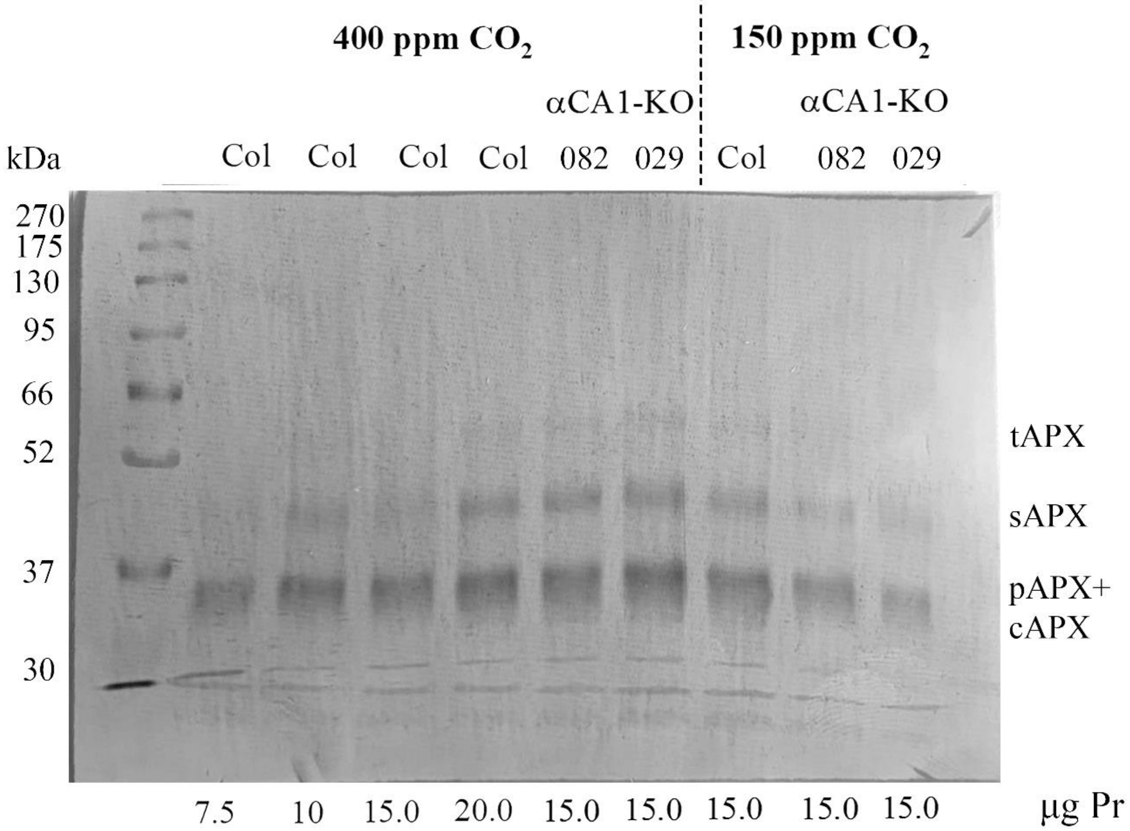

B

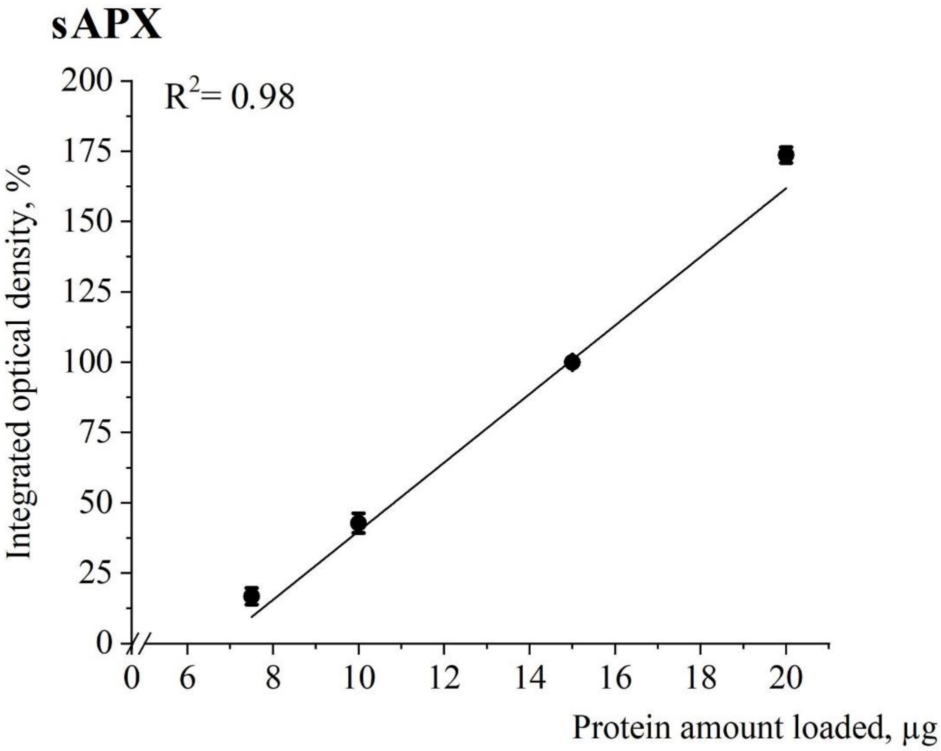

C

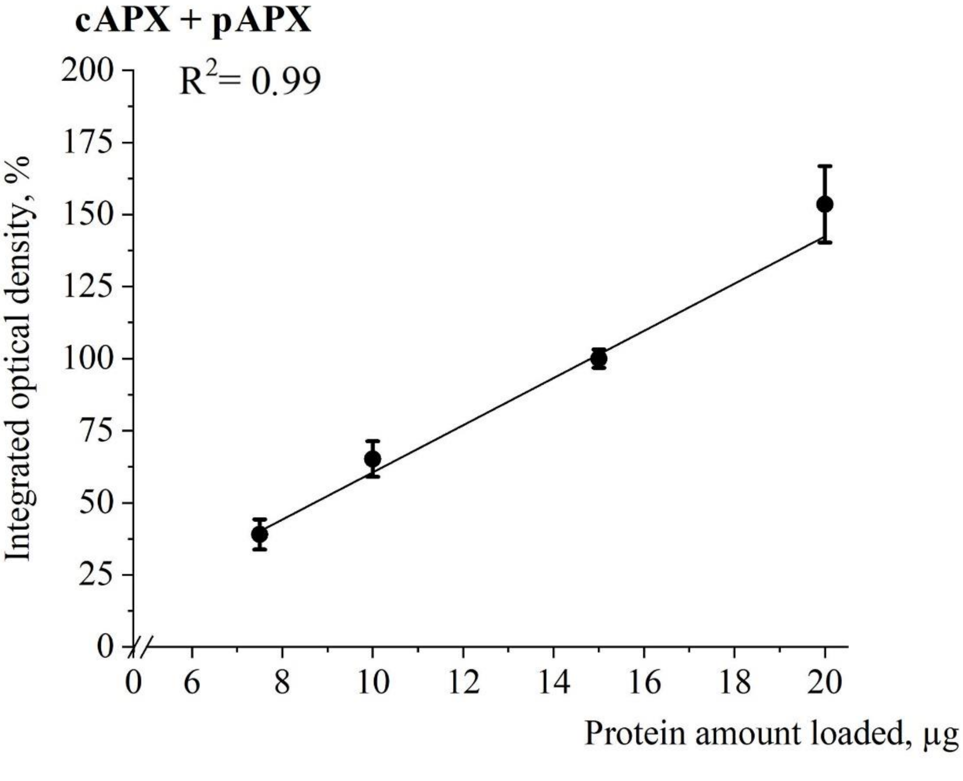

Figure S1. (A) Representative immunoblot image showing the abundance of ascorbate peroxidase (APX) isoforms corresponding to thylakoid ascorbate peroxidase (tAPX), stromal ascorbate peroxidase (sAPX), peroxisomal ascorbate peroxidase (pAPX), cytosolic ascorbate peroxidase (cAPX) in the leaves of the wild-type *A. thaliana* (Col) and αCA1-KO plants (082 and 029 lines). Figures B, C show the calibration lines for densitometric analysis of immunoblotting with antibodies against APX of the bands of sAPX and cAPX+ pAPX, respectively. Crude protein extracts from A. thaliana leaves were prepared using the modified

extraction method described in the Section 4.5. The calibration curve was generated using a protein preparation from Col plants grown at 450 ppm CO_2 (NCO_2). The integrated optical densities of immunoreactive bands were determined, and the protein amount of 15 µg Pr per lane was taken as 100%. The calibration points represent relative protein concentrations expressed as a percentage of this reference concentration. Data are presented as means ± SD. The solid lines represent linear regression within the linear dynamic range meanings indicated in the correspondent figure. The content of thylakoid APX was not analyzed because the signal for this APX isoform was too weak (Figure S1). For densitometric analysis, regions of interest (ROIs) were manually defined according to the expected migration position of each APX isoform and kept identical across samples; when neighboring bands were not fully resolved, the ROI encompassed their combined signal (cAPX + pAPX), and the bands were quantified together. For sAPX, which showed clearly separated bands, each band was evaluated separately using an individual ROI. Background signal was determined from an adjacent band-free area and subtracted from the integrated optical density.

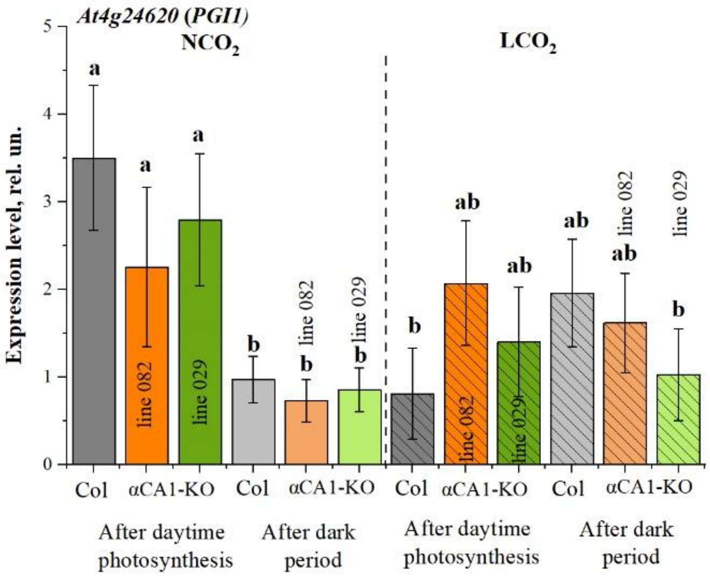

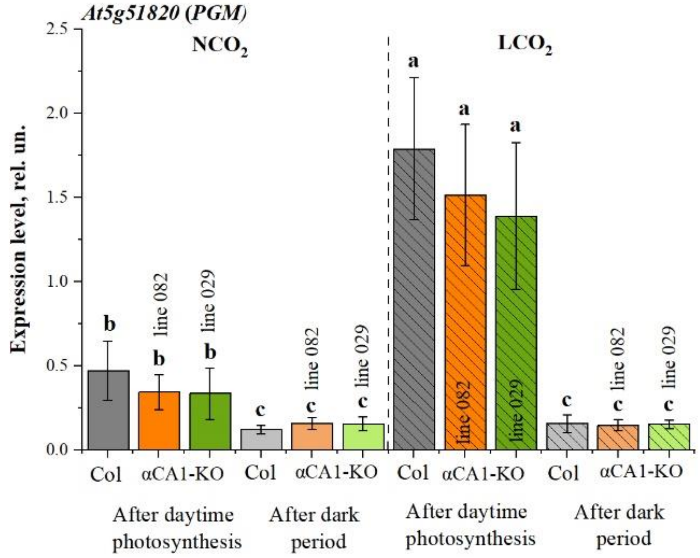

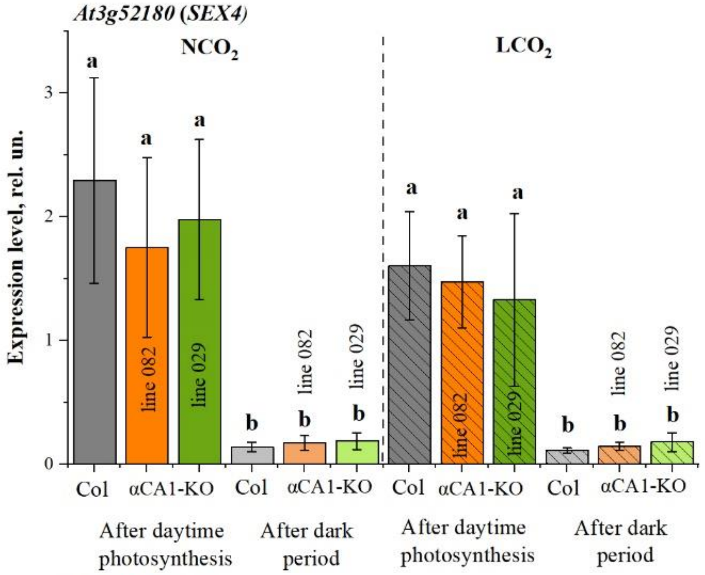
At3g52180 (SEX4)
NCO2
LCO2
Expression level, rel. un.
line 082
line 029
Col
αCA1-KO
After daytime photosynthesis
After dark period

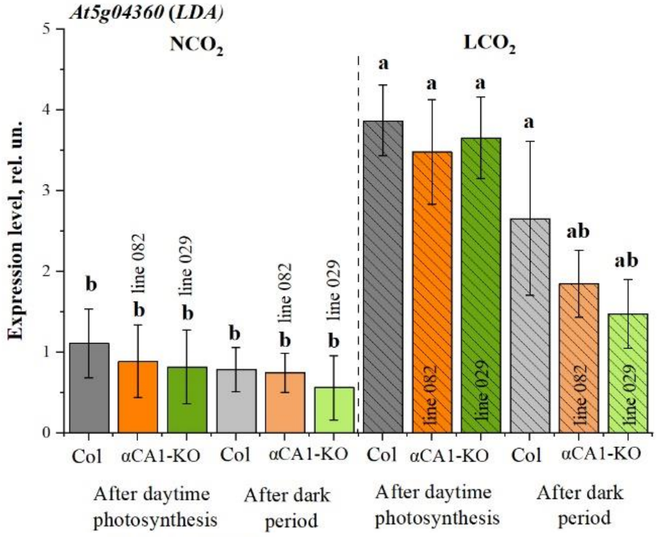
At5g04360 (LDA)
NCO2
LCO2
Expression level, rel. un.
line 082
line 029
Col
αCA1-KO
After daytime photosynthesis
After dark period

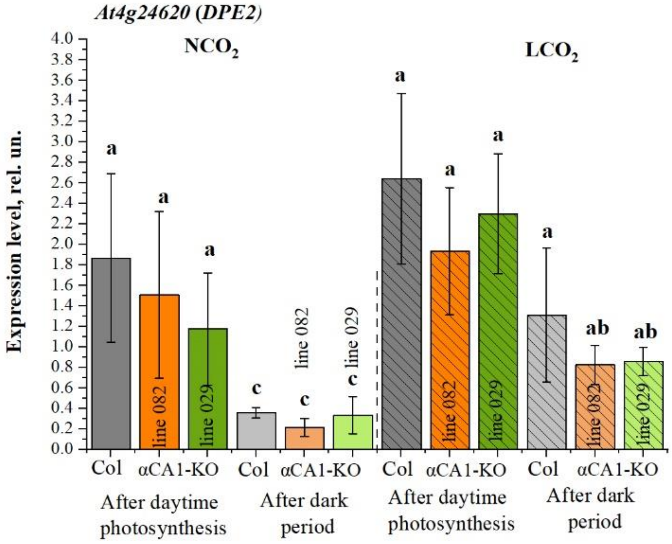
At4g24620 (DPE2)
NCO2
LCO2
Expression level, rel. un.
line 082
line 029
Col
αCA1-KO
After daytime photosynthesis
After dark period

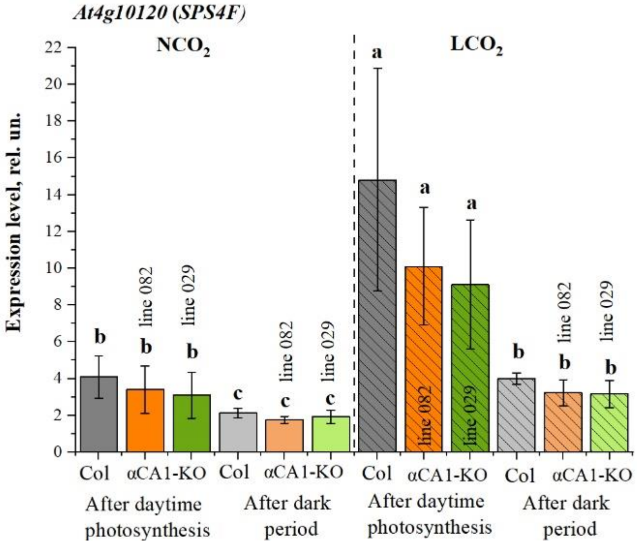

Figure S2. The expression level of the genes encoding the proteins involved in starch metabolism: phosphoglucose isomerase (*PGI1*), phosphoglucomutase (*PGM*), phosphoglucan phosphatase starch excess 4 (*SEX4*), α-1,6-glucosidase starch debranching enzyme limit dextrinase (*LDA*), 4-α-glucanotransferase disproportionating enzyme (*DPE*), sucrose phosphate synthase 4F (*SPS4F*) in the leaves of wild type *A. thaliana* (Col) and αCA1-KO plants (082 and 029 lines) after 16 h of darkness directly after turning on the light and after 8 h directly before turning off the light. Data were normalized for *Ubiquitin* gene expression. Values are means ± SD of three independent experiments. For each genotype and growth-CO_2 condition, 3 plants were sampled, with 2–3 leaves collected from each plant and pooled to obtain one biological sample. Each plant-derived sample was considered one independent biological replicate (total n = 9 across the three experiments). Three analytical replicates were performed for each biological sample. Statistical significance of genotype and growth-CO_2 effects was assessed by three-way ANOVA followed by the Holm–Bonferroni multiple-comparison test. Differences were considered statistically significant at $p < 0.05$. Letters above the data indicate statistically significant differences between experimental variants.

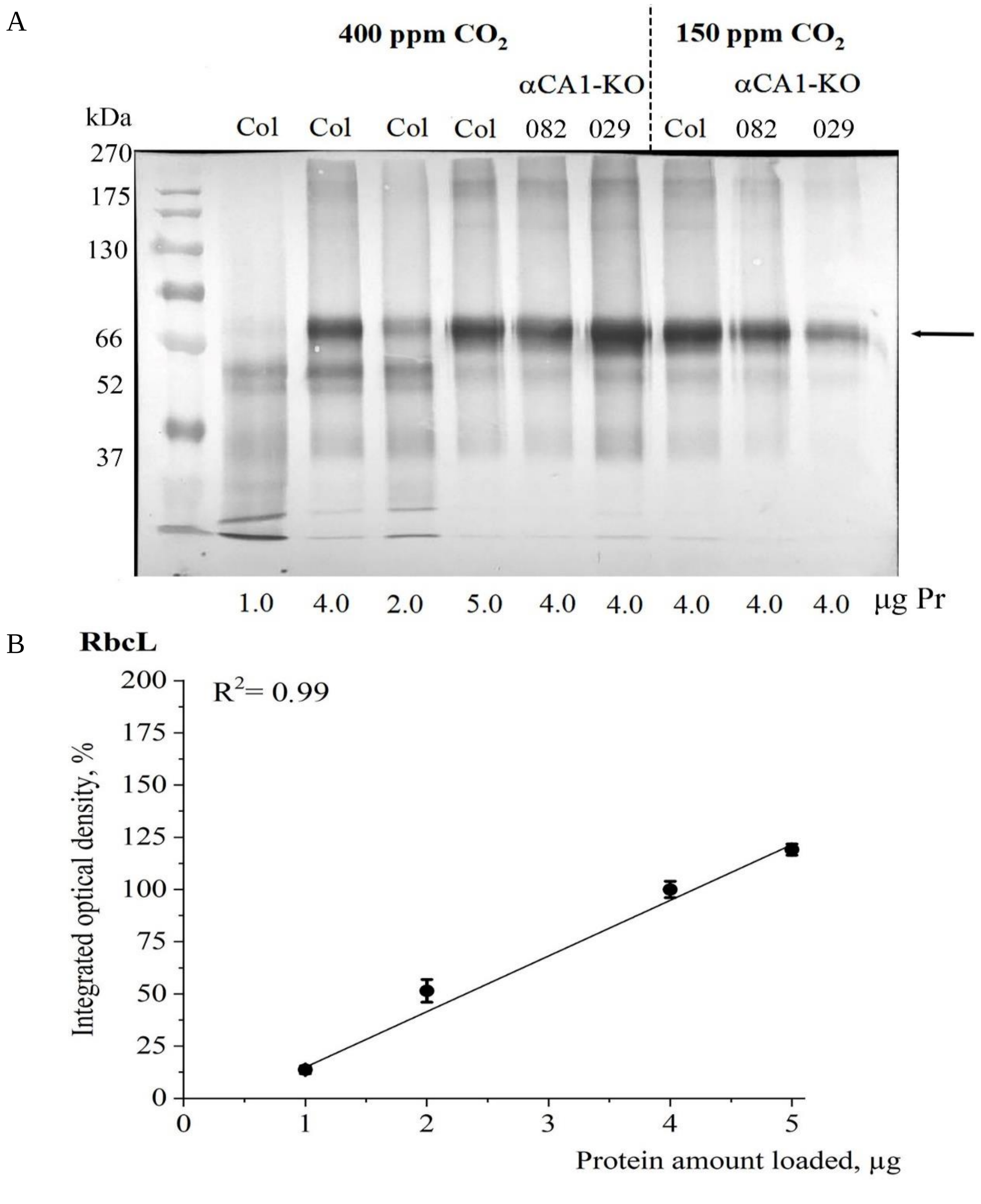
A
400 ppm CO_2
150 ppm CO_2
αCA1-KO
αCA1-KO
kDa
Col
Col
Col
Col
082
029
Col
082
029
270
175
130
66
52
37
1.0
4.0
2.0
5.0
4.0
4.0
4.0
4.0
4.0
μg Pr
B
RbcL
R^2= 0.99
Integrated optical density, %
Protein amount loaded, μg
0
25
50
75
100
125
150
175
200
0
1
2
3
4
5

C

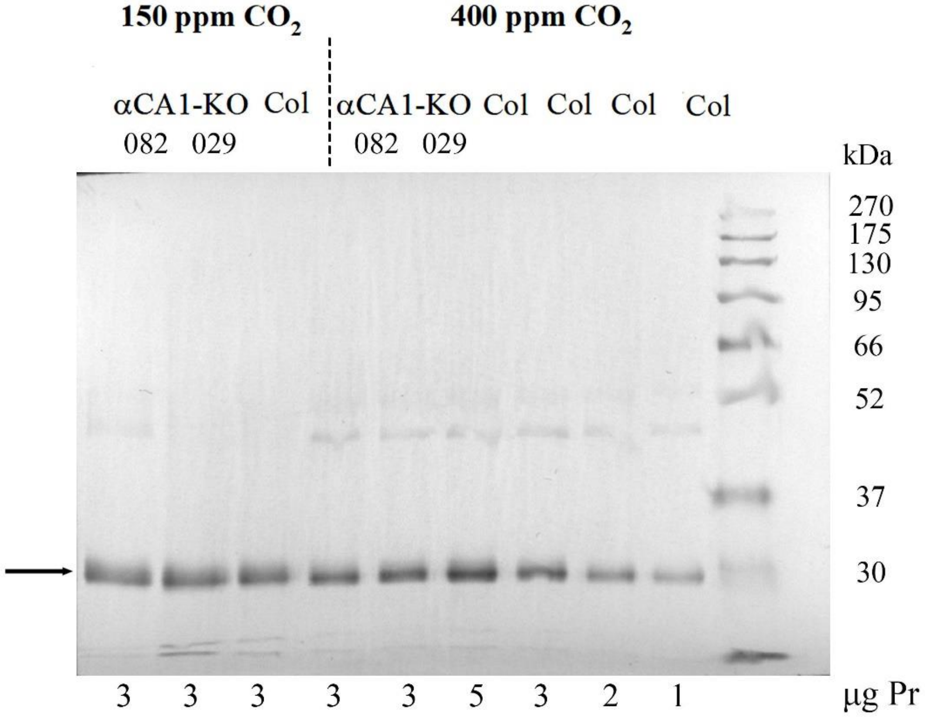

D

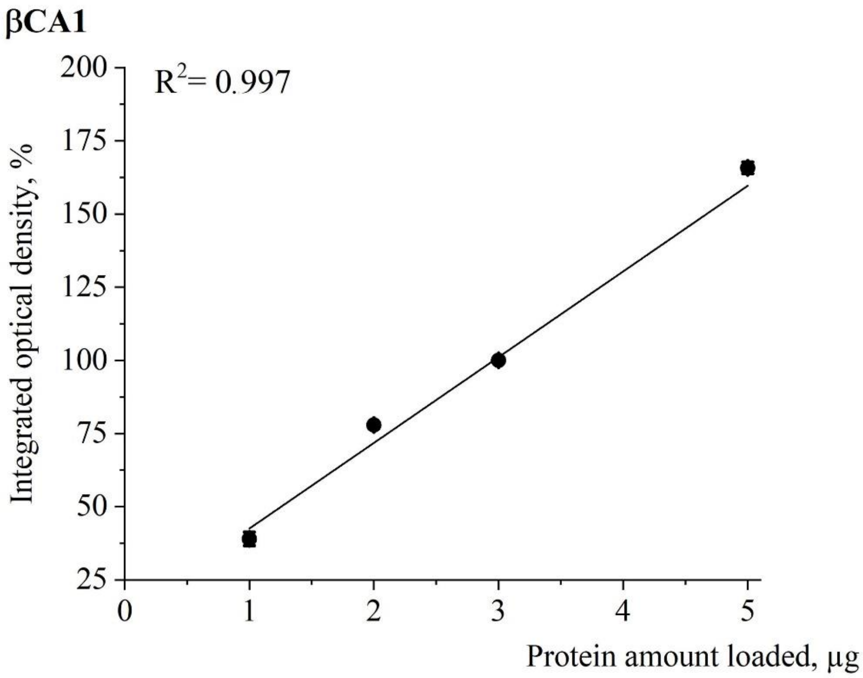

Figure S3. Representative immunoblot images showing the abundance of ribulose-1,5-bisphosphate carboxylase/oxygenase (RuBisCO) large subunit (RbcL) (A) and βCA1(C) (marked by arrows) in the leaves of wild type *A. thaliana* (Col) and αCA1-KO plants (082 and 029 lines). The concentrations of proteins in the preparations (µg Pr) on the lanes are indicated. Figures B, D show the calibration lines for densitometric analysis of immunoblotting with antibodies against RbcL and βCA1, respectively. Crude protein extracts from *A. thaliana* leaves were prepared using the modified extraction method described in the Section 4.5. The calibration curve was generated using a protein preparation from Col plants grown at 450 ppm CO_2 (NCO_2). The integrated optical densities of immunoreactive bands were determined, and the protein amount of 4 and 3 µg Pr per lane was taken as 100% for RbcL and βCA1, respectively. The calibration points represent relative protein concentrations expressed as a percentage of this reference concentration. Data are presented as means ± SD.

The solid lines represent linear regression within the linear dynamic range meanings indicated in the correspondent figure. For densitometric analysis, regions of interest (ROIs) were manually defined according to the expected migration position of each target protein and kept identical across samples. For βCA1 and RbcL the ROI encompassed the entire immunoreactive band. Background signal was determined from an adjacent band-free area and subtracted from the integrated optical density.

A B C

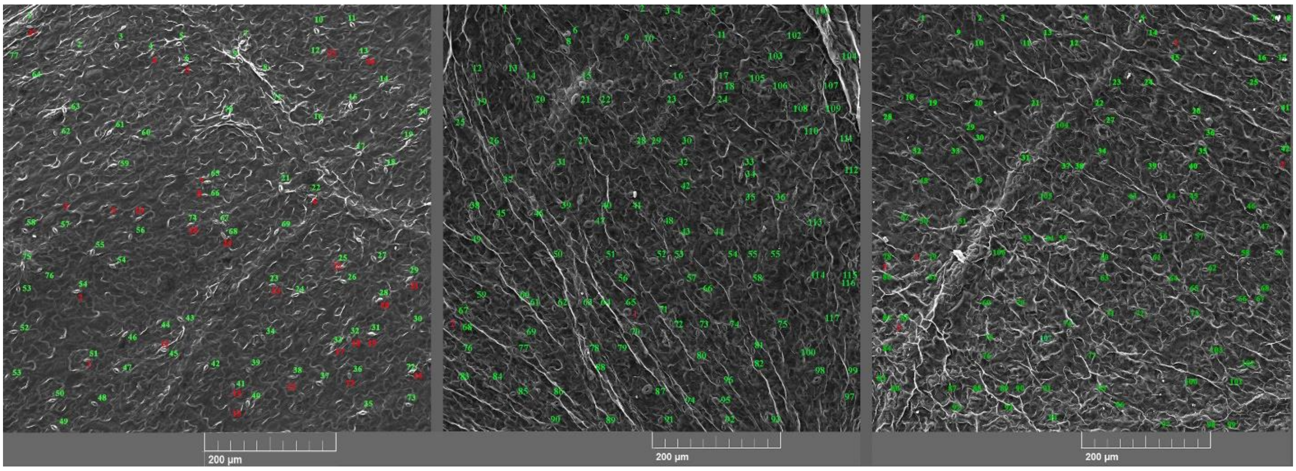

Figure S4. Representative image obtained using scanning electron microscope of the reverse leaf surface of *Arabidopsis thaliana* Col plants (A) and αCA1-KO plants (line 082 (B) and line 029 (C)). Green numbers indicate the stomata detected. Red numbers indicate the closed stomata. Instrument: TESCAN VEGA3 SEM.

A

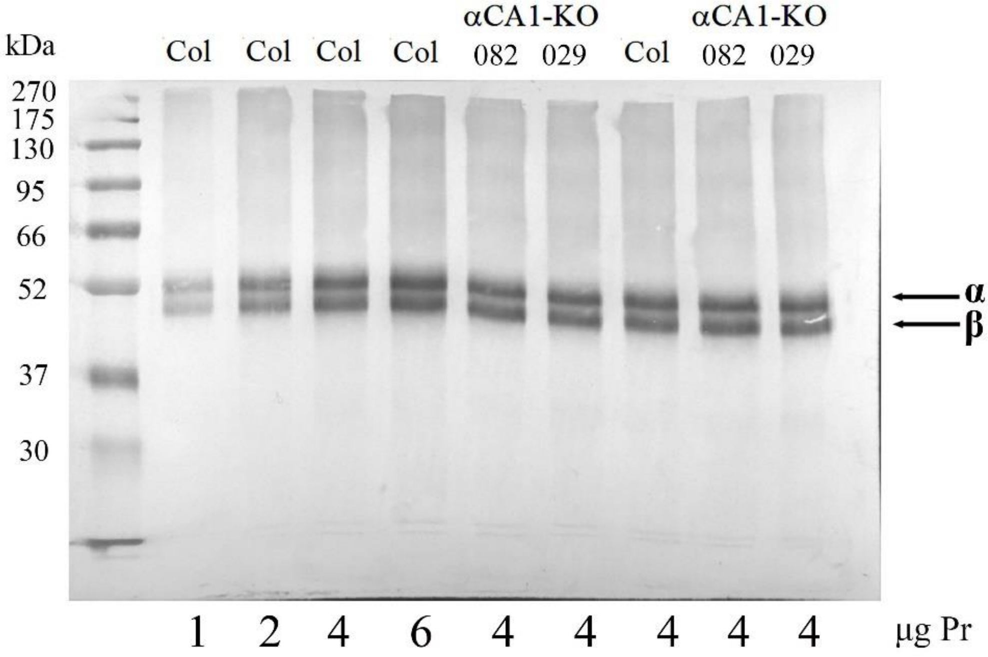

B

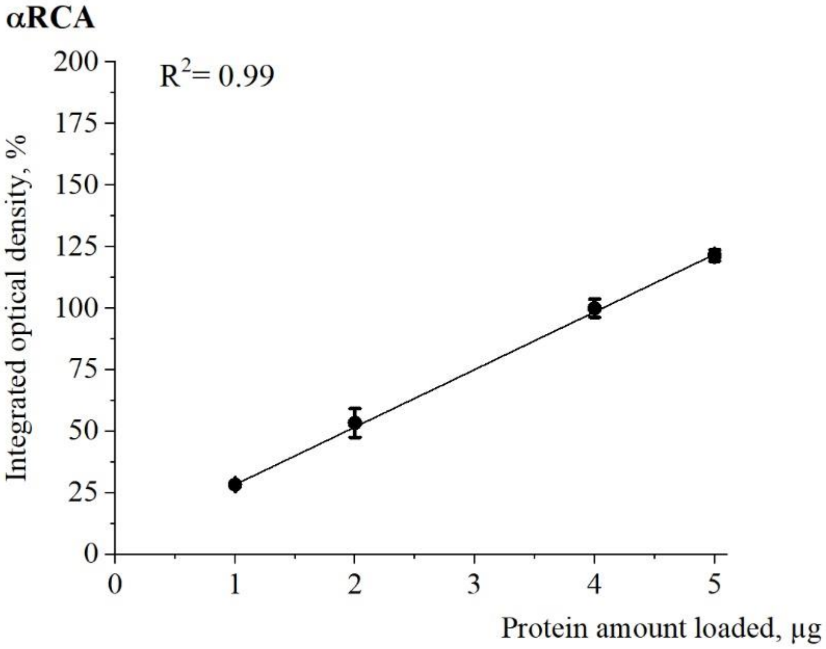

C

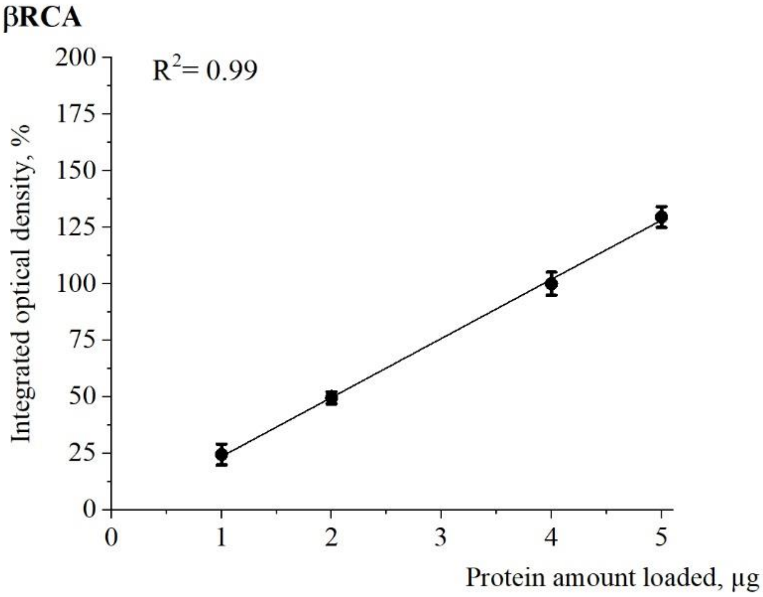

Figure S5. Representative immunoblot images showing the abundance of RuBisCO activase (RCA), α and β subunits (marked by arrows) in the leaves of wild type *A. thaliana* (Col) and αCA1-KO plants (082 and 029 lines) grown under NCO_2 conditions. The concentrations of proteins in the preparations (μg Pr) on the lanes are indicated. Figures B, C show the calibration lines for densitometric analysis of immunoblotting with antibodies against RCA, α and β isoforms, respectively. Crude protein extracts from *A. thaliana* leaves were prepared using the modified extraction method described in the Section 4.5. The calibration curve was generated using a protein preparation from Col plants grown at 450 ppm CO_2 (NCO_2). The integrated optical densities of immunoreactive bands were determined, and the protein amount of 4 μg Pr per lane was taken as 100%. The calibration points represent relative protein concentrations expressed as a percentage of this reference concentration. Data are presented as means ± SD. The solid lines represent linear regression within the linear dynamic range meanings indicated in the correspondent figure. For densitometric analysis, regions of interest (ROIs) were manually defined according to the expected migration position of each target protein and kept identical across samples. For αRCA, βRCA, which showed clearly separated bands, each band was evaluated separately using an individual ROI. Background signal was determined from an adjacent band-free area and subtracted from the integrated optical density.

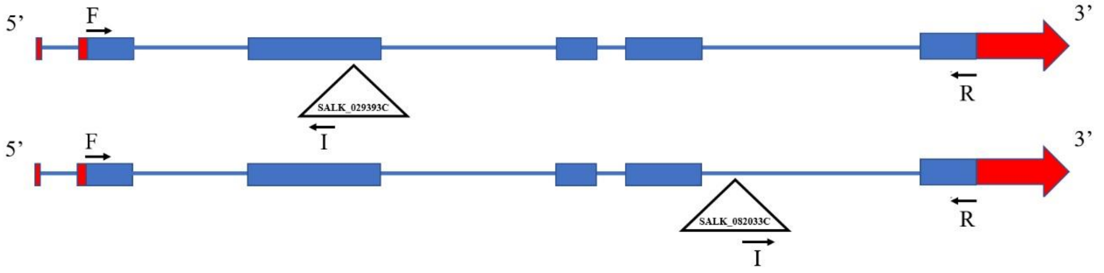

Figure S6. Location of the T-DNA insertion mutations in *αCA1* gene. The TDNA insertion in 029 line (SALK_029393C) is located in the third exon, the TDNA insertion (SALK_082033C) is located in the fifth intron of the αCA1 gene. Blue boxes represent exons, blue lines represent introns, red boxes represent 5’ UTRs, and red arrows represent 3’ UTRs along with the directionality of the genes. Black triangles represent T-DNA insertions. Black arrows represent locations of gene-specific primers. ‘F’ (5’ATGAAGATTATGATGATGATTAAGCTC3’) and ‘R’ (5’TTAATTGGGTTTTTCTTTTTGTTACC3’) are the forward and reverse primers used for genomic PCR and RT-PCR. ‘I’ (5'CCAAACTGGAACAACACTCAACCC3’) is the insert primer used for genomic PCR.

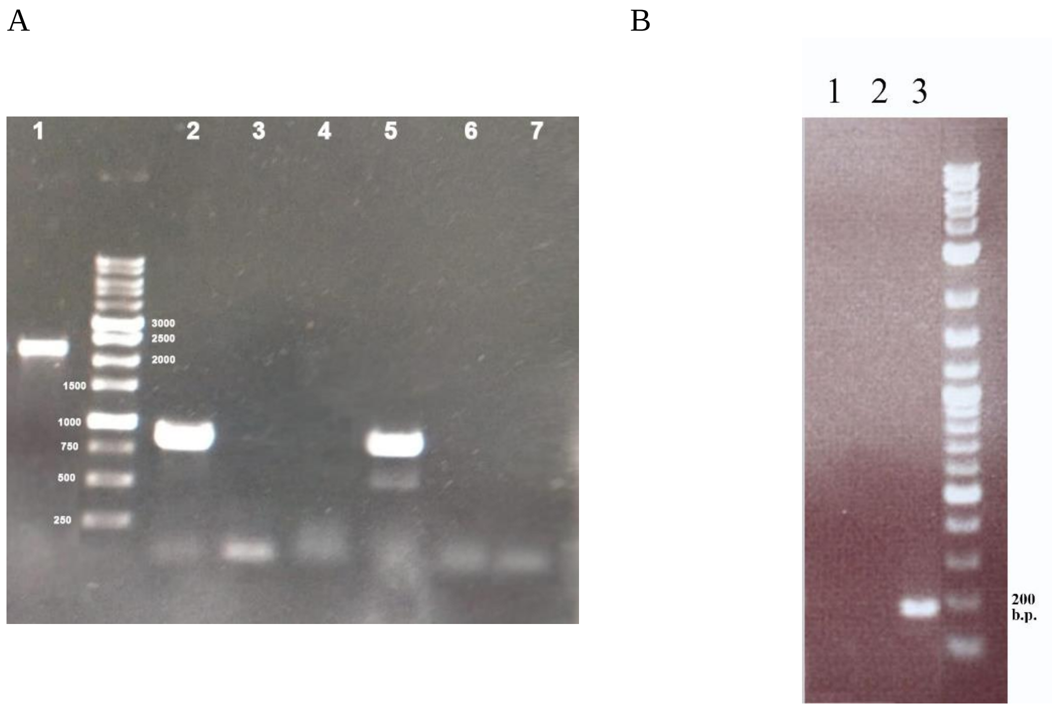

Figure S7. PCR check for the mutation.

(A) PCR was performed using PCR mixture 2X Blitz Mastermix (Belbiolab, Moscow, Russia) with DNA isolated from Col and mutant plants and primers complementary to DNA in positions marked in the Fig. S5. 1 – DNA from Col, primers F+R; 2 – DNA from 029 line, primers F+I; 3 – DNA from 029 line, primers R+I; 4 – DNA from 082 line, primers F+I; 5 – DNA from 082 line, primers R+I; 6 – DNA from 029 line, primers F+R; 7 – DNA from 082 line, primers F+R.

(B) PCR was performed with cDNA synthesized from RNA isolated from Col and mutant plants and primers complementary to exon regions with predicted molecular mass of PCR products 186 base pairs (F: 5’TGC TCA GAC AGA AGG AGT AGT GTT TG3’; R: 5’GTT TGT GAA GTA GTA TTC ACG GTG TAT TG3’). 1 – cDNA from 082 line, 2 – cDNA from 029 line, 3 – cDNA from Col.

Table S1. Primers used for qRT-PCR. F is the "forward" and R is the "reverse" primer.

Genes	Nucleotide sequences of primers	
At5g04360 (*ADP-glucose pyrophosphorylase* (*APS1*) gene)	F	5'GGTGACAATGTCAAGATCATAAACAGC3'
	R	5'AAAAAGCCATAAAGAAAACTTATTGATAGGGCA3'
At1g32900 (*Granule-bound starch synthase 1* (*GBSS*) gene)	F	5'TTGTGGACACTGTGAAAGATGGCT3'
	R	5'TCGCAGGTCCCTTCCAG3'
At4g25000 (*Endo-amylase α-amylase 1* (*AMY1*) gene)	F	5'ACATAGAATGGGGACTAAAAGAGAGCATC3'
	R	5'GCCTGAATAAGCTAAAGCAAAATTAGAAGGAAC3'
At5g48300 (*Phosphoglucomutase* (*PGM*) gene)	F	5'GAAAGAGTATTGGGCGACATACGGA3'
	R	5'ACTTCCATCAACAGGGTCTGTGTAT3'
At3g52180 (*Starch excess 4* (*SEX4*) gene)	F	5'GAC ATT GGA TGG GGA CAG AGG ATA C3'
	R	5'TTG TTG GAT CGT CCA CTA CTT TAG CG3'
At5g20280 (*Sucrose phosphate synthase 1* (*SPSA1*) gene)	F	5'TCGTATACAAGCTCTGAGGTATTTGTTCGT3'
	R	5'AGGAGATCACGTCGGTGAGAG3'
At4g10120 *(Sucrose phosphate synthase 4F* (*SPS4F*) gene)	F	5'AAGCACTCAGGTATCTTTCTATAAGGTGGG3'
	R	5'TCT CTT GTG GAA CTG CGT CTT CTC T3'
At2g40840 (*α-1,6-glucosidase Starch debranching enzyme* (*DBE2*) gene)	F	5'GGATATGATGGCTCTGAAAGAAGAGTACACTA3'
	R	5'TTG ATG TCT TCA CCA GAA ACA TTA GCA GG3'
At4g25000 (*α-1,6-glucosidase starch debranching enzyme Limit dextrinase* (*LDA*) gene)	F	5'TATACAGGAGCGAGTGCGATTTCATA3'
	R	5'TTTTAGAGCGGGACTGGGATATGAG3'
At4g24620 (*Phosphoglucose isomerase* (*PGI1*) gene)	F	5'CGGTGTGGAAGCTGGGAAAAA3'
	R	5'TGCTATCAGAACTCTGTCGTTTGC3'
At5g25760 (Arabidopsis *Ubiquitin* gene)	F	5'TGCTTGGAGTCCTGCTTGGA3'
	R	5'TGTGCCATTGAATTGAACCCTCT3'
